# Docosahexaenoic Acid-Driven Metabolic Reprogramming as an Attractive Anti-Infection Strategy to Potentiate β-Lactam Antibiotic Efficacy

**DOI:** 10.34133/research.0650

**Published:** 2025-03-28

**Authors:** Lei Xu, Sangyu Hu, Yuzhu Pei, Yutong Zhou, Xiuli Zhang, Linlin Ding, Minhe Cui, Yonglin Zhou, Xuming Deng, Zihao Teng, Jianfeng Wang

**Affiliations:** ^1^ State Key Laboratory for Diagnosis and Treatment of Severe Zoonotic Infectious Diseases, Key Laboratory for Zoonosis Research of the Ministry of Education, Institute of Zoonosis, and College of Veterinary Medicine, Jilin University, Changchun 130062, China.; ^2^ Jilin Mushuo Breeding Co., Ltd., Changchun 130052, Jilin, China.; ^3^ Key Laboratory of Ministry of Education for Conservation and Utilization of Special Biological Resources in the Western China, School of Life Sciences, Ningxia University, Yinchuan, China.

## Abstract

The emergence and prevalence of methicillin-resistant *Staphylococcus aureus* (MRSA) severely compromises conventional β-lactam antibiotics efficacy and poses an extensive global health challenge. Given the close relationship between docosahexaenoic acid (DHA) and metabolic alterations, this study aimed to reveal the novel function of DHA to potentiate β-lactam antibiotics activity through a lipid peroxidation mechanism. Additionally, DHA exhibited significant inhibitory effects on the catalytic function of β-lactamase through interactions with active residues. Herein, the dual-faceted mechanisms of perturbation of lipid metabolism and β-lactamase catalytic inhibition achieved the potentiated antibacterial efficacy of β-lactam antibiotics in combination with DHA against MRSA. Furthermore, to enhance the pharmacodynamic performance and stability of DHA, amoxicillin and DHA co-loaded nanoemulsions (Amo/DHA-NEs) were prepared via high-energy emulsification. Intriguingly, we found that Amo/DHA-NEs effectively rescued MRSA-induced infections in the murine infection models, as evidenced by the superior bacterial clearance and mitigated inflammation. Collectively, this work reveals a potentially exploitable link between DHA-driven metabolic reprogramming and β-lactams resistance, and we propose combination therapies of DHA and β-lactams targeting the emerging threat of MRSA infections.

## Introduction

Methicillin-resistant *Staphylococcus aureus* (MRSA) has emerged as a major zoonotic pathogen with public health and veterinary importance worldwide, possessing the characteristics of epidemiology and microbial pathogenicity [1]. Over the past decades, MRSA infections posed a broad spectrum of diseases involving skin and soft tissue infections, bacteremia, pneumonia, and mastitis [2]. The mechanisms underlying the emergence and development of antimicrobial resistance associated with MRSA commonly involve a complex system encompassing enzymatic inactivation, modifying the drug target sites, and enhancing efflux [3,4]. Given the challenges of antibiotic resistance brought by MRSA, traditional antibacterial agents and anti-infection strategies may become increasingly ineffective at alarming rates. Accordingly, combination therapy as an alternative and promising approach has attracted extensive and tremendous attention for managing the MRSA infections.

Long-chain polyunsaturated omega-3 fatty acids (n-3 PUFAs) including docosahexaenoic acid (DHA) are recommended as beneficial dietary supplements, which have shown the multifaceted impact of anti-inflammatory, antitumor, blood pressure and lipid metabolism regulation, and cognitive function improvement [5–7]. Nevertheless, the synergistic antibacterial activity of DHA and the underlying mechanisms remain elusive. Given that the iron-catalyzed peroxidation of PUFA has been proven to trigger a regulated cell death, ferroptosis in membrane phospholipids [8,9], these evidences suggested that PUFAs might be of great potential to mitigate infection by the destruction of bacterial lipid metabolism and iron homeostasis. Therefore, the close relationship between DHA and iron-dependent cell death characterized by lipid peroxidation inspires new insights into the mechanisms of the antimicrobial therapeutic potential of DHA.

Nanostructured systems, especially nanoparticles and the nano-emulsification technique, have been widely explored to surmount the challenges and issues of low bioavailability and bioactivity over the past few decades [10–12]. Recently, natural emulsifiers such as whey protein (WP) exhibit a wide range of activities, involving the prominent emulsification property, optimal amphipathy, and water solubility, in this sense, which has attracted increasing interest in the nanoemulsion preparation using WP as an emulsifier [13]. Peng et al. [14] reported on *Litsea cubeba* essential oil nano-emulsion using WP as the emulsifier through an ultrasonic-assisted method, enhancing its stability and bioactivity and resulting in superior bacteriostatic and antioxidant properties. Additionally, the formulation and development of DHA-loaded nanoparticles utilizing diverse delivery systems, involving low-density lipoprotein, zein, and resveratrol-stearate, also exerted prominent physiochemical properties and bioavailability, solidifying the efficacy as potential therapeutic strategies [15,16]. These findings highlight its applications and great potentials in biotechnological and pharmaceutical industries.

The characteristics of a double-edged sword are associated with the prospect of the widespread application of PUFAs. However, our understanding of the antimicrobial potentials of DHA and the underlying mechanisms are still not thorough. In this research, we revealed that the increased susceptibility to β-lactam antibiotics was elicited upon the exposure to the DHA (22:6n3). We proposed that DHA potentiated β-lactams activity by the regulation of bacterial metabolic plasticity. To expound this hypothesis, we investigated the potentials and mechanisms of DHA as a novel β-lactams adjuvant in tackling MRSA infections. These findings enlighten an exploitable link between DHA and antibiotics resistance, and reveal a novel function of DHA to potentiate β-lactam antibiotics efficacy through a lipid peroxidation mechanism.

## Results

### DHA potentiates antimicrobial activities of β-lactam antibiotics against MRSA

**Fig. 1.**
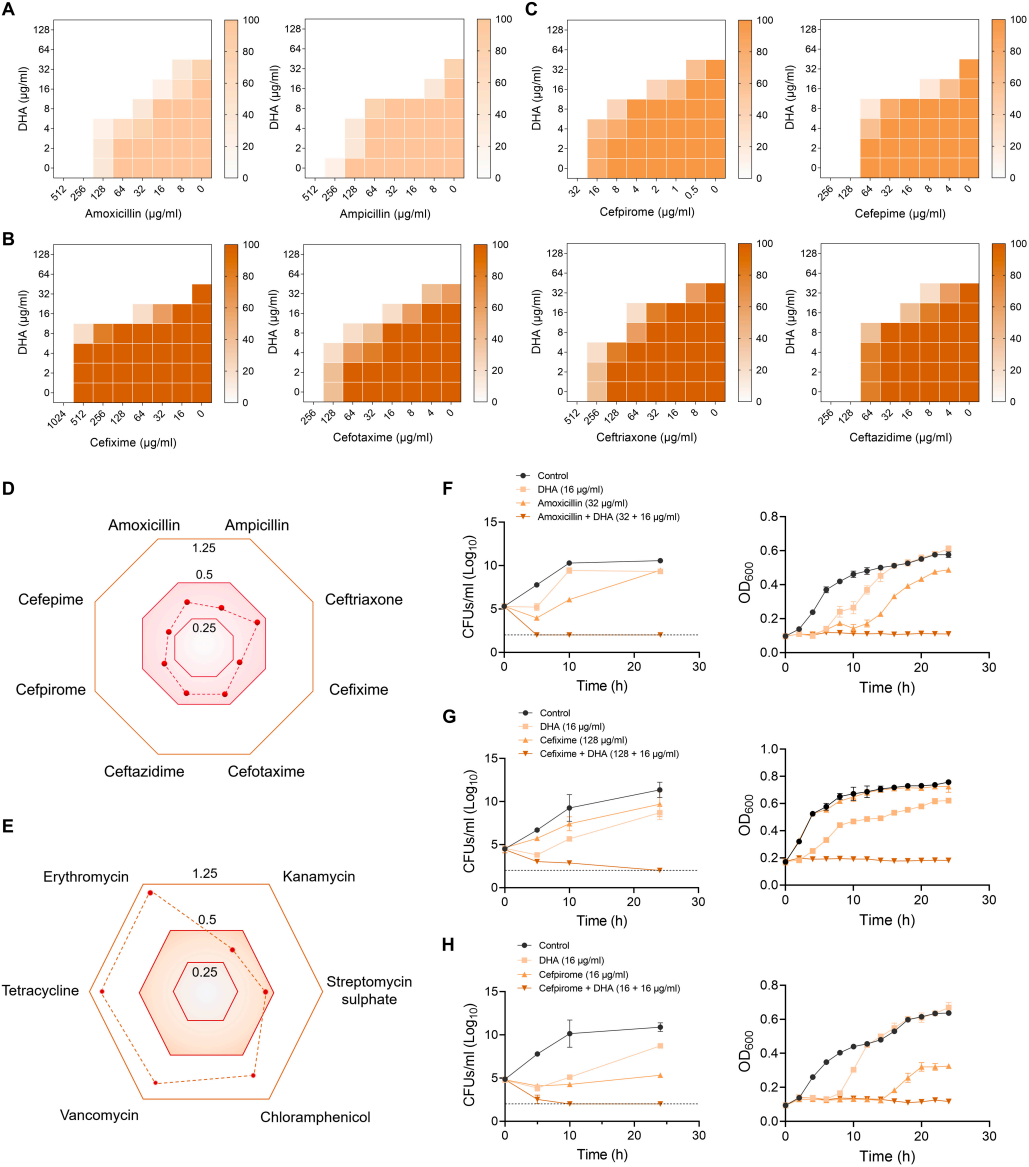
DHA potentiates β-lactam antibiotics susceptibility against MRSA. (A to C) Chequerboard broth microdilution assays of DHA in combination with penicillins (amoxicillin and ampicillin) (A) and the third- or fourth-generation cephalosporins (B and C) against MRSA. Dark regions represent the higher cell density and were shown with orange color (*n* = 5). Fractional inhibitory concentration indices (FICIs) for DHA combined with β-lactams (D) and diverse antibiotics (E) (*n* = 5). (F to H) Killing kinetics and growth curves of DHA with or without the representative β-lactam antibiotics (amoxicillin, cefixime, and cefpirome) (*n* = 3).

Synergistic combinations of DHA and β-lactam antibiotics against MRSA were assessed using a standard checkerboard assay, and interactions between DHA and β-lactam antibiotics were identified as synergistic with a fractional inhibitory concentration index (FICI) value of ≤0.5. Intriguingly, in comparison with amoxicillin or ampicillin monotherapy, combination treatment exhibited a noteworthy increase of antimicrobial efficacy (Fig. 1A). Specifically, DHA (16 μg/ml), in combination with third- or fourth-generation cephalosporins, including ceftriaxone, cefixime, cefotaxime, ceftazidime, cefpirome, and cefepime, displayed prominently synergistic activities against MRSA, as evidenced by 4- to 16-fold lower minimal inhibitory concentrations (MICs) (Fig. 1B to D). Interestingly, we found that DHA exerted an obvious synergistic antibacterial activity with β-lactams and aminoglycoside antibiotics (including kanamycin and streptomycin sulfate) (Fig. 1D and E). Next, we conducted a time-dependent killing curve to investigate their synergistic bactericidal activity. As revealed in Fig. 1F to H, the combinations of DHA and β-lactam antibiotics dramatically decreased bacterial counts, and finally thoroughly eradicated all *S. aureus* within 24 h postincubation, showing effectively synergistic potentials. Consistently, the results of the growth curve indicated that DHA plus β-lactam antibiotics (amoxicillin, cefixime, and cefpirome) showcased notable improvements in antimicrobial efficacies (Fig. 1F to H). Additionally, the disc diffusion method was employed to further expound the synergistic properties of DHA combined with amoxicillin, and as predicted in Fig. S1, the presence of DHA potently potentiated the amoxicillin activity in a concentration-dependent manner. Further, cell viability under the treatment of increasing concentrations of DHA with the presence or absence of amoxicillin was determined by lactate dehydrogenase (LDH) assay, and as indicated in Fig. S2, DHA monotreatment or in combination with amoxicillin exerted no safety concerns in cells. In summary, these results provided a potent basis and evidence for DHA to enhance the effectiveness of diverse β-lactam antibiotics.

### DHA boosts β-lactam antibiotics sensitivity in MRSA by a metabolic state reprogramming approach

**Fig. 2.**
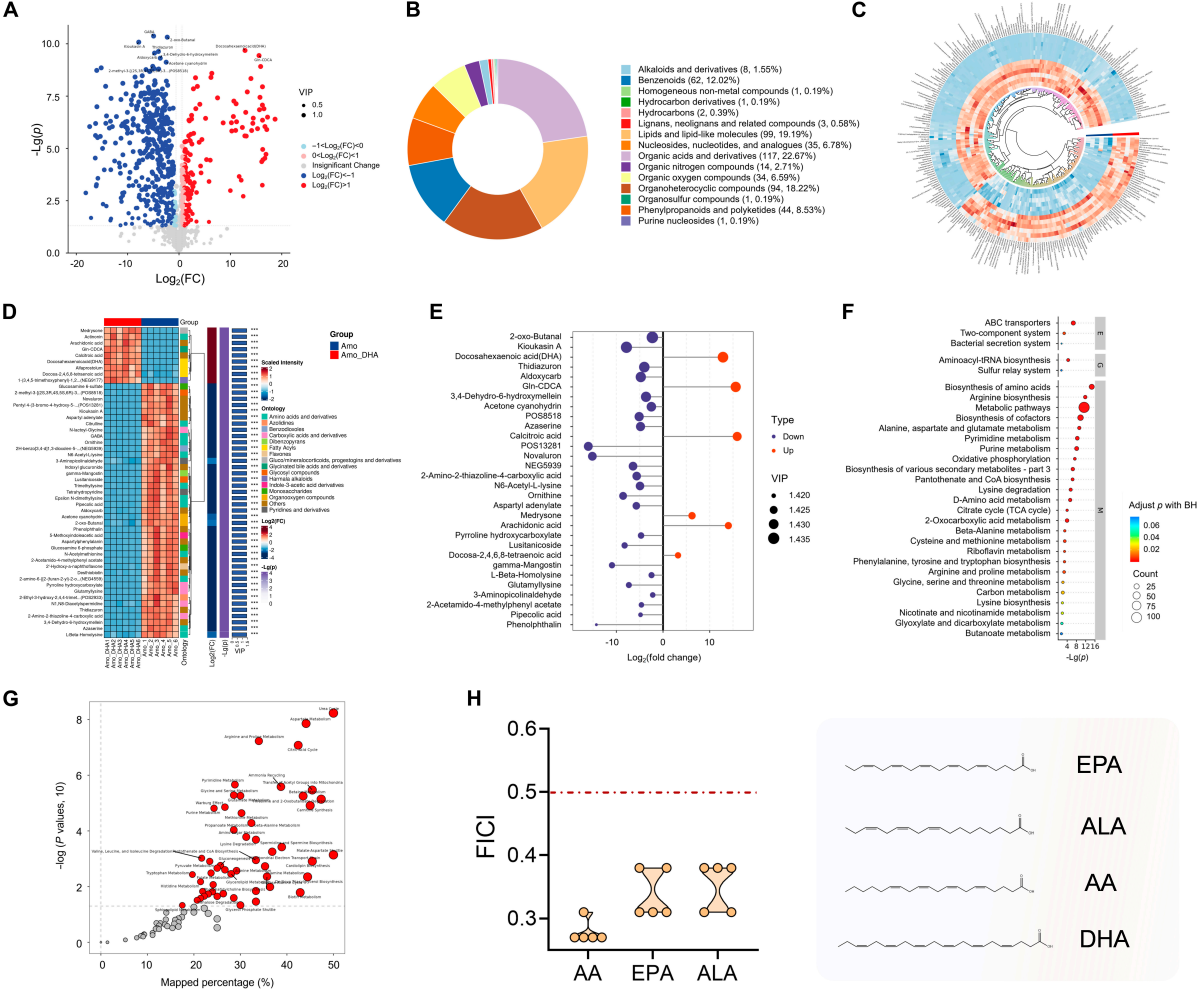
Metabolism reprogramming is crucial for the synergism of DHA and amoxicillin. (A) Volcano plots indicating the results of pairwise comparisons of differential metabolites in MRSA treated with amoxicillin alone or in combination with DHA. VIP, variable importance in projection; FC, multiple of the difference between groups. (B) The different metabolites of each comparison group were classified. (C and D) Circle heatmap and complex heatmap for hierarchical clustering analysis of differential metabolites between amoxicillin and combination treatment groups (*n* = 6). (E) Log2 (fold change) employed for the importance of differential metabolite analysis. Important metabolites were presented in red (up-regulation) or deep blue (down-regulation). (F and G) Kyoto Encyclopedia of Genes and Genomes (KEGG) and Human Metabolome Database (HMDB) database were used for metabolic pathway enrichment analysis. (H) Synergistic effects of long-chain polyunsaturated fatty acids (LC-PUFA) in combination with amoxicillin.

To elucidate molecular mechanisms of the susceptibility of DHA-mediated β-lactam antibiotics, we focused on the metabolism alteration in MRSA. We further analyzed the metabolite synthesis levels in bacterial cells treated with amoxicillin or in combination with DHA for underlying key mechanisms. Initially, distinct clusters of the combination treatment group compared with amoxicillin monotreatment were confirmed by principal component analysis (PCA), partial least squares discrimination analysis (PLS-DA), and orthogonal partial least squares discriminant analysis (OPLS-DA), which further revealed the reliable data quality (Fig. S3A to C). As predicted in metabolomics results, the distribution of different metabolites was presented by volcano plots, revealing the distinct metabolite levels between amoxicillin monotreatment and the combination treatment of amoxicillin and DHA (Fig. 2A). According to the structure and function of metabolites, the differential metabolites of each comparison group were classified and statistically analyzed. As revealed in Fig. 2B, the differential metabolites were mainly linked to the organic acids and derivatives (22.67%), and lipids and lipid-like molecules (19.19%), suggesting that lipid metabolism plays a key role in synergistic activity of DHA against MRSA. Moreover, we conducted an initial analysis among the differential metabolites in bacterial cells (Fig. 2C to E). The pathway enrichment analysis in Fig. 2F and G further indicated that these differential metabolites were mainly enriched in metabolic pathways, oxidative phosphorylation and central carbon metabolism (TCA cycle), and nitrogen metabolism (biosynthesis of amino acid, arginine biosynthesis, purine metabolism, and alanine, aspartate, and glutamate metabolism). These results prompted that the synergistic activity of combination therapy was associated with remodeling of bacterial metabolism, focusing on energy metabolism and lipid homeostasis. PUFAs were reported to react readily with molecular oxygen to form a lipid peroxyl radical and trigger a chain reaction of lipid peroxidation [9]. Given that PUFA-induced lipid peroxidation is closely associated with iron-dependent cell death, we speculated that the synergistic activity of DHA in combination with β-lactams might be attributed to the remodeling of lipid metabolism. Thus, to elucidate whether PUFA-driven lipid peroxidation was positively correlated with the potentiation of amoxicillin activity in *S. aureus*, the checkerboard assay was conducted. As revealed in Fig. 2H and Fig. S4A, PUFAs including arachidonic acid (AA), eicosapentaenoic acid (EPA), and alpha-linolenic acid (ALA) treatment effectively boosted amoxicillin activity against MRSA, which further reinforced the conjecture that antibiotic susceptibility is largely associated with PUFA-induced lipid peroxidation. Moreover, the increased DHA content in the combination group shown in Fig. S4B revealed that DHA might effectively integrate into bacterial membranes or bacterial cells for their synergistic antibacterial effect. In tandem, the increase of phosphatidylethanolamine PE (P-18:0/20:4) in the combination group, as markers related to lipid peroxidation, suggested the significance of iron homeostasis in the resensitization of bacteria to amoxicillin (Fig. S4C). Collectively, these findings suggested that the synergistic effect of DHA and amoxicillin is largely associated with the bacterial lipid peroxidation mechanism.

### DHA triggers metabolic perturbation and oxidative damage

**Fig. 3.**
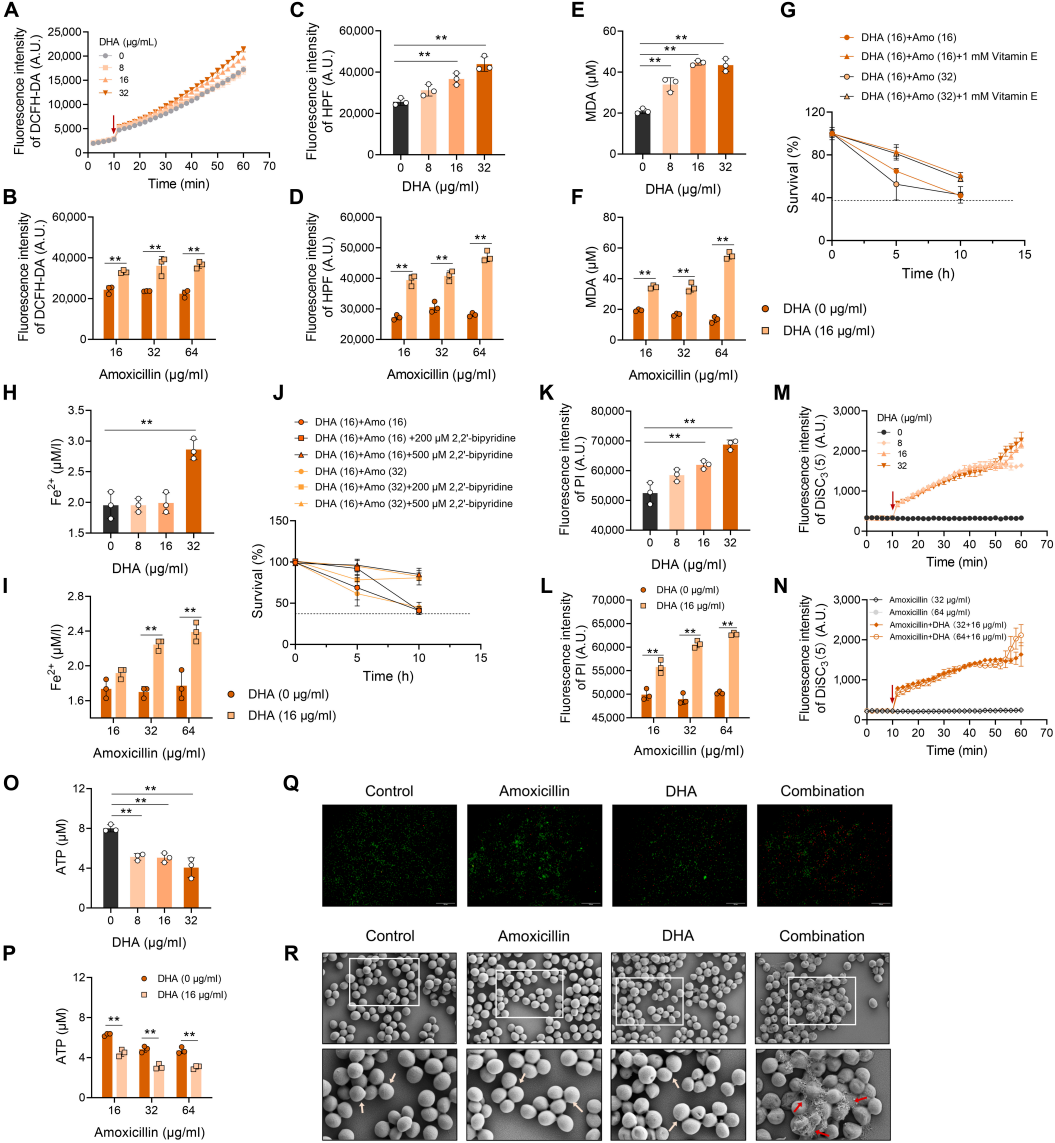
DHA enhances amoxicillin activity through a lipid peroxidation mechanism. Oxidative damage was determined by the reactive oxygen species (ROS) (A and B) and hydroxyl radicals formation (C and D), using the fluorescent probes DCFH-DA and hydroxyphenyl fluorescein (HPF). (E and F) Quantification of malondialdehyde (MDA) contents after DHA or amoxicillin monotreatment, or their combinations. (G) *S. aureus* was treated with DHA in combination with amoxicillin, in the presence or absence of 1 mM antioxidant vitamin E. (H and I) Intracellular ferrous iron under the treatment of DHA, amoxicillin alone, or their combinations. (J) Moreover, the synergistic activity of DHA in combination with amoxicillin was effectively counteracted by the addition of membrane-permeable iron chelator 2,2′-bipyridine. The membrane permeability (K and L) and membrane potential (M and N) in MRSA treated with the increasing concentrations of DHA or in combination with amoxicillin. (O and P) The intracellular ATP levels of *S. aureus* under the indicated treatments. (Q) The living (green fluorescence) and dead (red fluorescence) status of bacteria with the treatment of DHA alone or in combination with amoxicillin. Moreover, SEM observation was employed to identify the morphological changes of MRSA with the presence and absence of DHA and/or amoxicillin (R). Data are shown as means ± SD; *n* = 3 biological replicates. **P < 0.01.

We next sought to depict whether lipid peroxidation could account for the effective synergism between DHA and β-lactam antibiotics. To determine the oxidative stress level, the intracellular ROS levels under exposure to DHA ranging from 0 to 32 μg/ml alone, or DHA at a concentration of 16 μg/ml in combination with amoxicillin, were determined using a fluorescent probe, 2′,7′-dichlorodihydrofluorescein diacetate (DCFH-DA). As depicted in Fig. 3A and B, DHA monotreatment or combined with amoxicillin potently promoted the intracellular ROS accumulation compared with the control or amoxicillin alone, respectively. Further, the hydroxyl radical that caused deleterious oxidative damage to DNA, proteins, and membrane lipids was increased by DHA monotreatment or combined with amoxicillin (Fig. 3C and D). Importantly, the level of the lipid peroxidation product malondialdehyde (MDA) was thereupon boosted after exposure to combination treatment of DHA with indicated concentrations of amoxicillin (Fig. 3E and F), indicating that lipid peroxidation plays a critical role in its combined antibacterial activity. Moreover, Fig. 3G shows that the addition of α-tocopherol (vitamin E) as an antioxidant blocking lipid peroxidation observably reduced their synergistic bactericidal activity. These results underpin a major contribution of lipid peroxidation in the synergistic effect of DHA. Consistently, the transcriptions of *bsaA_2*, *katA*, *sodA*, and *sodM*, known to regulate the catalase and superoxide dismutases in *S. aureus*, were repressed by the combination therapy, compared with the amoxicillin monotreatment (Fig. S5). To better understand the nature of cell death induced by DHA administration, we further investigated the iron homeostasis in *S. aureus* [17–19]*.* It was noted that treatment with the excess DHA or in combination with amoxicillin resulted in a conspicuous enhancement on the intracellular ferrous iron contents (Fig. 3H and I), suggesting that the labile iron pool (LIP) sensitizes cells to ferroptosis-like cell death in *S. aureus.* Importantly, the addition of 2,2′-bipyridine, a ferrous iron chelator, exerted a blunted synergistic efficacy, in comparison with DHA plus amoxicillin treatment (Fig. 3J). In aggregate, these findings underscored that the addition of DHA facilitated ferroptosis-like cell death in MRSA, with the characteristics of lipid peroxidation and iron homeostasis disruption, and thereby potentiating the existing β-lactam antibiotic efficacy.

It is reported that PUFAs play crucial roles in cellular membrane permeability and fluidity [20]. As depicted in Fig. 3K and L, the membrane permeability of bacterial cells with the presence or absence of DHA/amoxicillin monotreatment or the combinations was determined using the propidium iodide (PI) dye, and as predicted, after exposure to DHA alone or combined with amoxicillin, the cell membrane permeability was significantly enhanced. Protons are pumped across the cellular membrane through the electron transfer chain (ETC), generating a proton gradient for adenosine triphosphate (ATP) synthesis [21]. Moreover, the fluorescence intensity of the DiSC_3_(5) probe was increased after the treatment of diverse concentrations of DHA, suggesting the disruption of membrane potential (ΔΨ), and in tandem, our results showed that the combination treatment of DHA and amoxicillin exerted a prominent potentiation on the fluorescence value of DiSC_3_(5) (Fig. 3M and N). Additionally, the intracellular ATP levels were decreased (Fig. 3O and P), indicating that excessive accumulation of DHA may destroy the activity of ETC. Further, Live/Dead cell staining results depicted in Fig. 3Q revealed that compared with DHA or amoxicillin treatment alone, proportion of dead cells labeled by PI was observably boosted after the combined treatment of DHA with amoxicillin. Scanning electron microscopy (SEM) was employed to assess the membrane integrity of bacterial cells, and as observed in Fig. 3R, DHA in combination with amoxicillin (64 μg/ml) triggered lytic cell death, culminating in cell membrane swelling and rupture. These results concluded that the cellular membrane integrity was destroyed by the combination treatment, accompanied by the alteration of cell membrane permeability and membrane potential disruption.

### DHA drives inhibition of β-lactamase activity

**Fig. 4.**
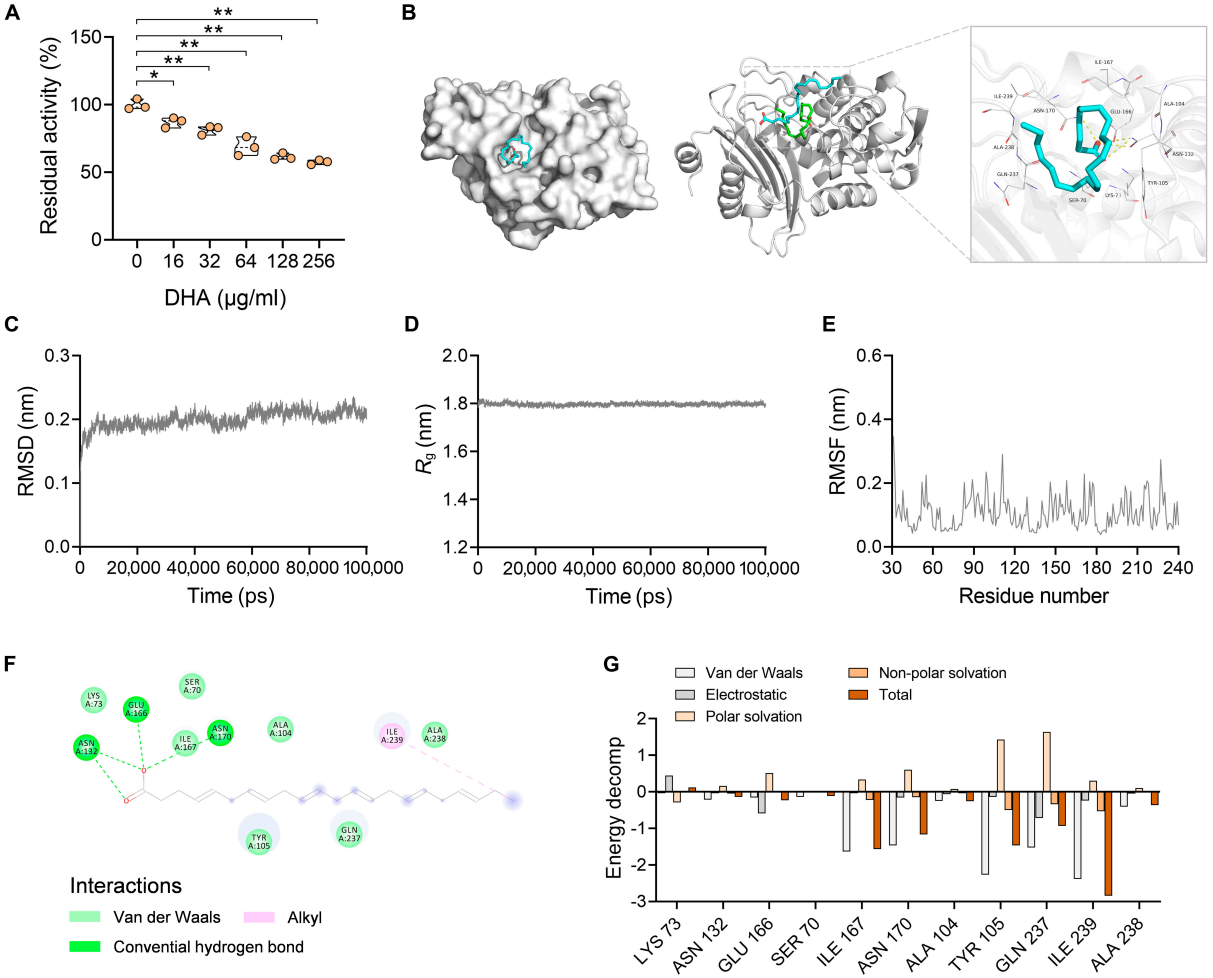
DHA effectively inhibits the hydrolytic activity of β-lactamase. (A) The β-lactamase enzyme produced by MRSA could hydrolyze β-lactam antibiotics. Nevertheless, β-lactamase activity is suppressed by the treatment of increasing concentrations of DHA. (B) The stable 3D structure of DHA/β-lactamase complex obtained through molecular dynamics simulations. The RMSD (C), *R*_g_ (D), and RMSF (E) plots of the DHA/β-lactamase complex. (F and G) The critical residues and binding energy decomposition for β-lactamase protein interacting with the ligand DHA were monitored throughout the simulation. *P < 0.05; **P < 0.01.

A well-known paradigm of β-lactam resistance is to produce β-lactamases responsible for catalyzing the opening and hydrolysis of the β-lactam ring of β-lactam antibiotics. Next, to further confirm the efficacy of DHA in the mechanism of β-lactamases, here we measured the ability of β-lactamase derived from *S. aureus* on the catalytic proficiency toward the hydrolysis of nitrocefin with the presence or absence of increased concentrations of DHA. As expected, DHA was capable of inhibiting the enzymatic activity of β-lactamase at the concentrations ranging from 16 to 256 μg/ml (Fig. 4A). To further explore the underlying mechanisms of DHA on the inhibition of β-lactamase, the molecular dynamics simulations were carried out. The simulated system interacted by the DHA and β-lactamase is shown in Fig. 4B. To assess the stability and binding of the DHA–β-lactamase complex, additional parameters involving root-mean-square deviation (RMSD), root-mean-square fluctuation (RMSF), and radius of gyration (*R*_g_) throughout the simulation were also determined. The RMSD between the protein–ligand complex, as a consequential indicator of conformational stability, provided insights into the equilibration status of protein. As shown in Fig. 4C, the RMSD value of β-lactamase protein indicated elevated conformational stability of the protein–ligand complex. Consistently, the similar trends were observed in the *R*_g_ simulation results (Fig. 4D). Additionally, RMSF is utilized to present details of the fluctuations in amino acid residues within the simulation runs, suggesting enhanced residue dynamics, rendering protein flexibility and dynamics (Fig. 4E). Importantly, Fig. 4F displays the cumulative energy involving van der Waals forces, hydrogen bond, and alkyl interactions, calculated for the interaction between the protein–ligand complex, showcasing the strong interactions of DHA with the key amino acid residues of β-lactamase (i.e., LYS73, ASN132, GLU166, SER70, ILE167, ASN170, ALA104, TYR105, GLN237, ILE239, and ALA238). Next, decomposition of the binding energy in the DHA–β-lactamase complex was depicted in Fig. 4G. Briefly, stable protein–ligand complex interactions including van der Waals interactions, electrostatic, polar solvation, nonpolar solvation, and total contributions of the amino acid residues were evidenced by molecular mechanics/generalized born surface area (MM/GBSA) calculations to estimate the residues surrounding the binding site and analyze the ligand-binding affinities based on free energy calculations. Collectively, these results provided comprehensive evidences for the interactions between the DHA–β-lactamase complex, thereby exerting significant inhibition on β-lactamase activity.

### The preparation and characterization of amoxicillin and DHA dual drug-loaded nanoemulsions

**Fig. 5.**
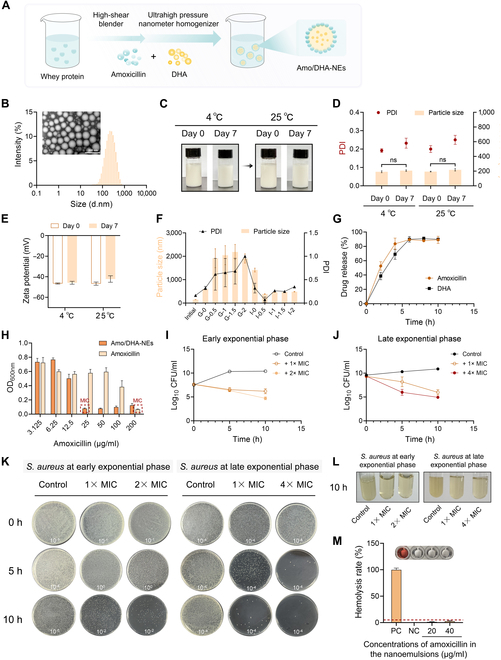
Preparation and characterization of the nanoemulsions Amo/DHA-NEs. (A) The preparation program of Amo/DHA-NEs. (B) TEM images of emulsions. Gross observation (C), particle size distribution (D), and zeta potentials (E) were conducted to determine the storage stability of Amo/DHA-NEs at 4 or 25 °C (*n* = 3). (F) The particle size distribution and polymer dispersion index (PDI) of Amo/DHA-NEs after 0, 0.5, 1, 1.5, and 2 h of digestion in the simulated stomach or intestine digestion, respectively (*n* = 3). (G) Cumulative release studies in amoxicillin and DHA dual drug-loaded nanoemulsion. (H) The MICs values against MRSA were determined by detecting the absorbance values of the cultures at 600 nm using a microplate reader. (I and J) The bactericidal effects of Amo/DHA-NEs against MRSA at early exponential phase and late exponential phase were measured by time-dependent killing analysis. (K and L) The representing coating plates and bacteria grew images of MRSA after the different treatment were shown. The dilution ratio used in the plate count method were recorded in white on the representative plates. (M) Hemolysis rates under the different treatment. Experiments are representative of at least 3 biological replicates and all data were presented as mean ± SD. ns, *P* > 0.05.

Amoxicillin and DHA dual drug-loaded nanoemulsions (Amo/DHA-NEs) were further developed for augmenting uptake and enhancing oral bioavailability, and the synthetic procedure diagrams are shown in Fig. 5A. The transmission electron microscope (TEM) image in Fig. 5B revealed that the aspect and nanostructure of Amo/DHA-NEs were formed with the characteristics of near-spherical distributions and the particle sizes were in the range of 100 to 200 nm. To expound the storage stability of Amo/DHA-NEs, the appearance of emulsions displayed inapparent alteration (Fig. 5C). Further, after 7 days of storage, the average particle size and zeta potential (ZP) of Amo/DHA-NEs within the temperature range of 4 to 25 °C exhibited minor fluctuations, indicating that the emulsions were relatively stable (Fig. 5D and E). Collectively, these combined results confirmed the successful preparation and outstanding physical stability of Amo/DHA-NEs.

Nanocarriers, such as nanoemulsions (NE), have been proven to enhance the bioavailability by potentiating its solubility and intestinal permeability and absorption of active substances [22,23]. Furthermore, the effects of in vitro gastrointestinal digestion on Amo/DHA-NEs were investigated, and as revealed in Fig. 5F, the substantial alterations in both particle size and polydispersity index (PDI) were observed during the period of simulated gastrointestinal digestion. As predicted, acidic conditions in the gastric environment altered the structural properties of Amo/DHA-NEs, resulting in larger aggregate formation. After intestinal digestion, the particle size and PDI of Amo/DHA-NEs were obviously decreased, indicating that the nanoemulsion system underwent enzymatic degradation under simulated intestinal conditions, and subsequently, new micelles and vesicles were formed to increase the solubilization and transcellular absorption of bioactive compounds (Fig. 5F), consistent with previous research findings [14]. Further, Amo/DHA-NEs possessed high EE% with 92.45% ± 1.34% (DHA) and 80.91% ± 4.65% (amoxicillin). The drug release profile showed that the release of amoxicillin and DHA from the nanoemulsion reached approximately 90% within 6 h (Fig. 5G). These results inferred that Amo/DHA-NEs could respond to the intestinal environment to trigger the drug release and reduce the unnecessary exposure.

For in vitro antibacterial effects evaluation, Fig. 5H indicated that the dual-loaded nanoemulsions Amo/DHA-NEs exerted superior antibacterial activity against MRSA, compared to amoxicillin monotreatment. Furthermore, Amo/DHA-NEs displayed obvious bactericidal effects against MRSA at both early exponential phase (Fig. 5I) and late exponential phase (Fig. 5J). Decreased bacteria grew and bacterial colonies were observed in the Amo/DHA-NEs treatment group (Fig. 5K and L). Additionally, the increasing concentrations of Amo/DHA-NEs exhibited almost no obvious hemolysis (less than 5%), confirming great hemocompatibility (Fig. 5M). Collectively, these results revealed that amoxicillin and DHA dual drug-loaded nanoemulsions Amo/DHA-NEs provided a prospective and achievable therapeutic strategy for treating MRSA infections.

### In vivo safety evaluation and synergistic antimicrobial activity of DHA in a mouse systemic infection model

To further characterize the therapeutic potential of Amo/DHA-NEs, we investigated the in vivo toxicity, as illustrated in Fig. S6A. Moreover, Fig. S6B showed that the visual images of major organs including mice liver, kidney, spleen, lung, heart, and intestine exhibited no obvious damage under the experimental conditions. Furthermore, mice body weight and the values of hematological parameters, including white blood cell, neutrophil count, lymphocyte count, red blood cell, hemoglobin, and hematocrit, showed no significant difference among the diverse groups (Fig. S6C to H). These findings concluded that Amo/DHA-NEs elicited no obvious toxicity in vivo under the experimental conditions.

**Fig. 6.**
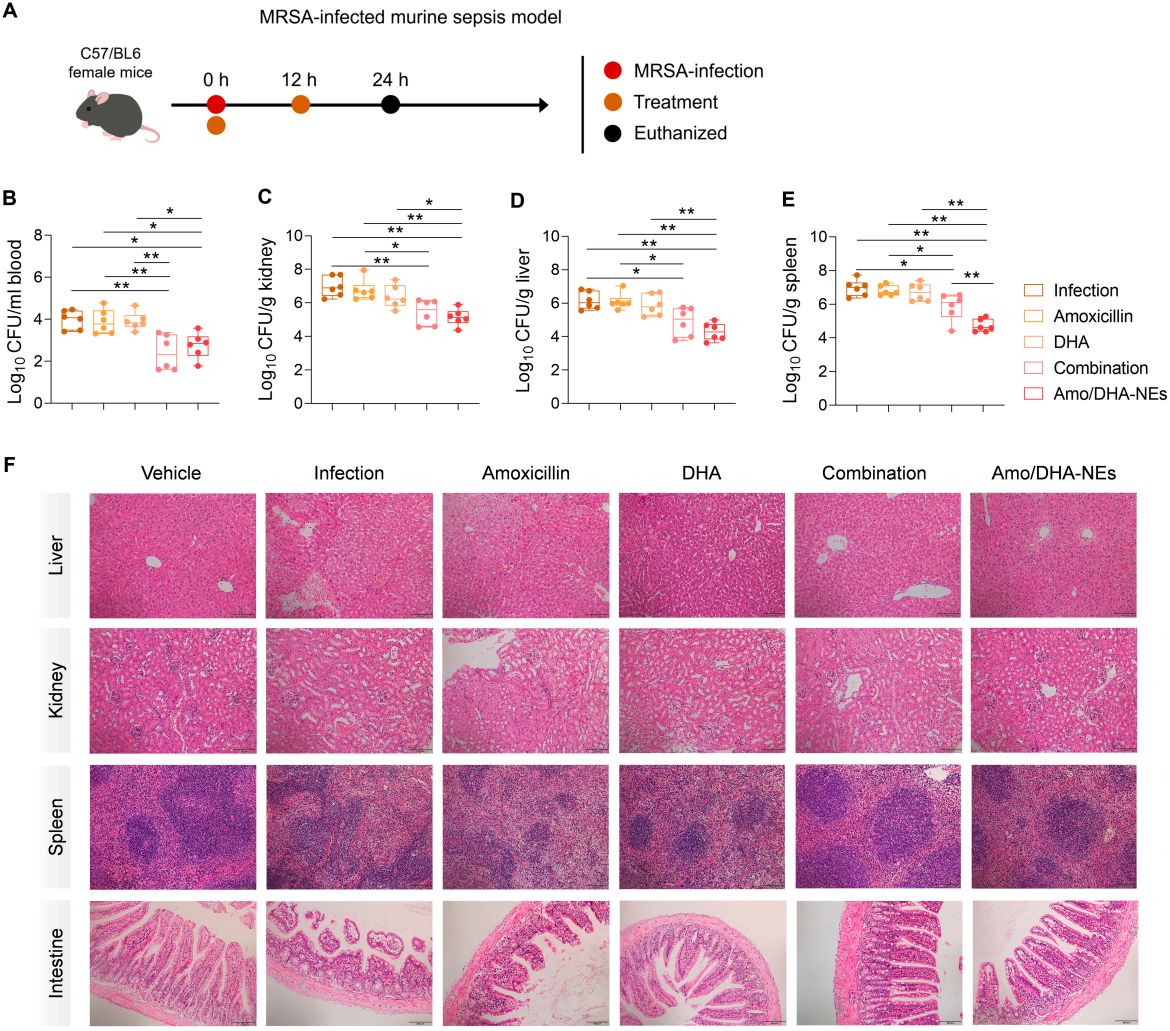
In vivo therapeutic efficacy evaluation in a mouse systemic infection model. (A) Schematic illustration of experimental protocol for therapeutic efficacy evaluation in vivo. (B to E) Bacterial load in the blood, kidney, spleen, and liver in different treatment groups in a mouse systemic infection model (*n* = 6). (F) Histopathological assessment of the liver, kidney, spleen, and intestine. Scale bar, 100 μm. *P < 0.05; **P < 0.01.

Furthermore, a murine systemic infection model induced by the intraperitoneal injection of MRSA was established to test the synergistic effect of DHA in combination with amoxicillin (Fig. 6A). The bacterial colony counts in the bloods, kidneys, spleens, and livers of mice in both the combination treatment group and the Amo/DHA-NEs group were observably decreased compared with the MRSA infection group or amoxicillin-treated mice (Fig. 6B to E). In particular, Amo/DHA-NEs eradicated bacteria more effectively in spleens (Fig. 6E).

Hematoxylin–eosin (H&E) staining and histological analysis revealed that the MRSA infection group displayed obvious hemorrhage, edema, vacuolar degeneration, and inflammatory infiltration in hepatocytes. Kidney tissues in the infection group showed extensive tubular degeneration and necrosis and focal hyperemia. The unclear boundary between the red pulp and white pulp of the spleen and villi fracture and thinning of the intestinal wall were also observed in the MRSA-infected mice. In contrast, these pathological characteristics were markedly alleviated by the combination treatment or Amo/DHA-NEs therapy (Fig. 6F and Fig. S7). In conclusion, these results suggested that Amo/DHA-NEs possess remarkable therapeutic efficacy, owing to their characteristics of alleviating overwhelming MRSA infections both in vitro and in vivo, whereas further studies should be assessed to explore the efficacy on a wider range of clinical infections.

### Anti-infection and anti-inflammatory action of DHA in combination with amoxicillin treatments for acute MRSA pneumonia therapy and MRSA-induced mastitis in mice

**Fig. 7.**
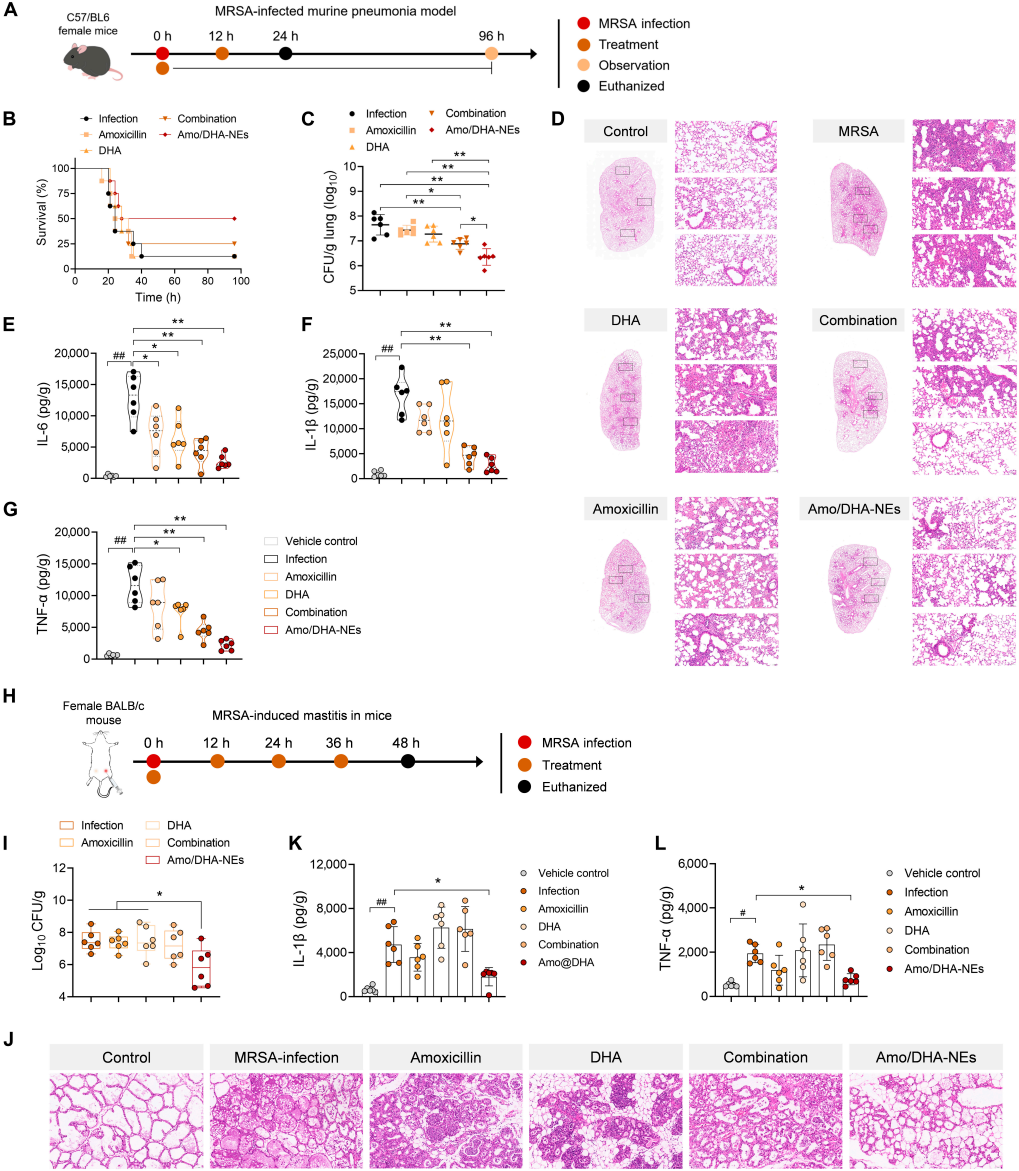
Synergistic effect of DHA in combination with amoxicillin in MRSA-infected pneumonia model and mastitis in mice. (A) Schematic illustration of experimental protocol for the establishment of a mouse model of acute MRSA pneumonia. (B) Survival curves of MRSA-infected pneumonia mice under the different treatment were monitored for 96 h postinfection (*n* = 8). (C) Bacterial load in the lung tissues of MRSA-infected mice (*n* = 6). (D) Histopathological observation of lungs after different treatments using H&E staining. Scale bar, 50 μm. Moreover, the inflammatory responses were determined by the detection of IL-6 (E), IL-1β (F), and TNF-α (G) levels in lungs (*n* = 6). (H) Experimental protocol for the MRSA-induced mastitis model under the different treatment. (I) Mammary bacterial burden in the MRSA infection mice mastitis model (*n* = 6). (J) Representative mammary H&E-stained images and the relevant histological scores from different treatment groups. Scale bar, 50 μm. IL-1β (K) and TNF-α (L) contents in mammary homogenate from the diverse groups were determined using ELISAs (*n* = 6).

We further assessed the therapeutical effect of Amo/DHA-NEs in a mouse model of acute MRSA pneumonia (Fig. 7A) and a MRSA-induced mouse mastitis model (Fig. 7H). As presented in the mice survival curve, it was found that in the infection group, 12.5% of MRSA pneumonia mice survived within 96 h postinfection; in contrast, the survival rate in the DHA and amoxicillin combination group or the Amo/DHA-NEs treatment group was markedly enhanced to 25% and 50%, respectively (Fig. 7B). Consistently, the lower colony-forming unit (CFU) burden in the lungs of the combination treatment group was displayed, in comparison with the MRSA infection group or amoxicillin monotherapy (Fig. 7C). Importantly, acute MRSA pneumonia mice by the Amo/DHA-NEs treatment showed the lowest bacterial loads in the lung tissues among all the mice under different treatments (Fig. 7C). Furthermore, H&E staining results revealed that hemorrhage and necrosis in pulmonary interstitial blood vessels and inflammatory cell infiltration were notably elevated in the acute MRSA pneumonia mice (MRSA infection group), whereas after the DHA + amoxicillin combination or the nanoemulsion Amo/DHA-NEs treatments, the pathological lesions in the lung tissues were observably alleviated, characterized by the depressed inflammatory exudates, cell infiltrates and congestion, and hemorrhage in the alveolar space (Fig. 7D). To investigate the effects of the combination or nanoemulsion Amo/DHA-NEs treatment on the inflammation level of mice, lung tissue homogenates in the diverse groups were collected to determine the proinflammatory cytokine tumor necrosis factor-α (TNF-α), interleukin (IL)-1β, and IL-6 levels via enzyme-linked immunosorbent assays (ELISAs). As depicted in Fig. 7E to G, the DHA + amoxicillin combination and Amo/DHA-NEs treatment groups exerted obviously reduced inflammatory cytokine levels of IL-1β, IL-6, and TNF-α in lungs, highlighting the efficacious anti-inflammatory effects of combination therapy. To sum up, these results further revealed that the combination of DHA and amoxicillin, especially the nanoemulsion Amo/DHA-NEs therapy, exerted remarkably synergistic antibacterial activity in vivo.

Moreover, the synergistic effect of DHA in combination with amoxicillin in the MRSA-induced mouse mastitis model remains unknown, and the whole experimental scheme for this study is shown in Fig. 7H. The consumption of DHA/amoxicillin-loaded nanoemulsion (Amo/DHA-NEs) observably decreased mammary bacterial burden compared with DHA or amoxicillin monotherapy upon the MRSA-infected mammary gland (Fig. 7I). Further, histological staining revealed that inflammatory cell infiltration and barrier damage caused by MRSA infection were observed in the infection samples or amoxicillin/DHA monotreatment samples; in contrast, Amo/DHA-NEs treatment prominently alleviated mammary injury (Fig. 7J). Furthermore, MRSA-infected mice, upon the treatment of Amo/DHA-NEs, exerted decreased contents of proinflammatory markers, including IL-1β and TNF-α in the mammary gland, contrasting with the MRSA infection group (Fig. 7K and L). These data collectively indicated that the treatment of nanoemulsion Amo/DHA-NEs alleviates *S. aureus*-induced mastitis in mice, showcasing notable and promising therapeutic potentials in effective mastitis therapy.

## Discussion

The dissemination and evolution of MRSA pose a tremendous challenge in clinical practices, and alarmingly, such crisis is seriously threatening the convenient therapeutic options in clinical settings and paralyzing the worldwide health system. The recent evidences underscored the prodigious morbidity and mortality associated with MRSA co-infections in conjunction with COVID-19 patients, and the growing antibiotic resistance among MRSA strains during the COVID-19 pandemic underscored an urgent and challenging issue to develop feasible approaches and strategies [24,25]. Despite increasing interests in funding novel antimicrobial development, the multifaceted impacts of a sparse drug pipeline and stagnant drug discovery severely limit clinical treatment therapeutic choices in the post-antibiotic era. In contrast, combination therapy to restore or enhance the antimicrobial activity of existing antibiotics proved promising in broadening their clinical applications to combat drug-resistant bacterial infections [26–28]. In this manner, we probed the synergism between DHA and β-lactams to overcome antibiotics resistance. Accurately deciphering the mechanisms underlying the synergistic efficacy of DHA in combination with β-lactams could present and strengthen a theoretical basis for enhancing the effectiveness of antibiotics therapy.

Accumulating evidences support the crucial role of bacterial metabolism in anti-infection therapy, which was identified as a potential target for antimicrobial development [29,30]. A recent work revealed that PUFAs, with a low host toxicity, enhanced the efficacy of aminoglycosides by the disruption of *S. aureus* membranes [20]. Additionally, the lipid peroxidation involving oxidation of AA to reactive electrophiles was revealed as the mechanism underlying the antimicrobial properties of AA [31]. These combined results of the studies inspired us to investigate the combination treatment-driven metabolic reprogramming for novel therapeutic strategies to combat MDR bacterial infections. Encouragingly, our findings proposed a novel combination therapy with DHA to potentiate β-lactam antibiotics activities through a lipid peroxidation mechanism, which may provide a promising antibacterial approach and a potential advantage of eradicating MRSA. In the current study, we revealed the beneficial role of DHA to potentiate β-lactam antibiotics against MRSA through a metabolism regulation angle. Indeed, despite the fact that ferroptosis-like cell death was proven to occur through excessive peroxidation of PUFAs, further studies are warranted to clarify the intrinsic effects of DHA on the regulation of iron metabolism.

DHA is highly susceptible to oxidation accompanied by the characteristics of intense odor and poor water solubility, which limit biomedical applications in the advancement of anti-infection therapy. Considering that the delivery of DHA to internal sites of infection is limited by the host mechanisms of DHA uptake and usage leading to low bioavailability, we further prepared Amo/DHA-NEs by high-energy methods, characterized by great physicochemical stability. Recently, accumulating evidences suggest that pharmaceutical nano-formulations showcase the potentials of therapeutic application to fundamentally alter the pharmacokinetics profile and tissue distribution, enhance drug solubility and intracellular bioavailability, and coordinate drug interaction for synergistic action [32–34]. Liu et al. [16] found that the bioavailability of DHA-loaded nanoparticles based on zein and PLGA (ZPDNPs) was 4.2- to 5.6-fold higher in comparison with free DHA. As revealed, our results unveiled dual DHA and amoxicillin-loaded nanoemulsions with the characteristics of high drug load, good stability, and being easily accessible exerted efficacious therapeutic potentials in vivo, as evidenced by a significant decrease in bacterial load and effective alleviation of pathological lesions. Importantly, the formation of nanoemulsions with a small particle size contributes to penetrate the cell membrane or intercellular space to reach infection sites after oral administration and thereby effectively improves the bioavailability of the drug. Notably, in this study, therapeutic efficacy of Amo/DHA-NEs was superior to free amoxicillin or the combination treatment in both MRSA-infected murine pneumonia model and mastitis in mice, which might profit from effectively improved drug bioavailability. Collectively, this study innovatively presented a dietary therapeutic strategy based on DHA and amoxicillin dual drug-loaded nanoemulsion formation to synergistically control the survival of MRSA.

**Fig. 8.**
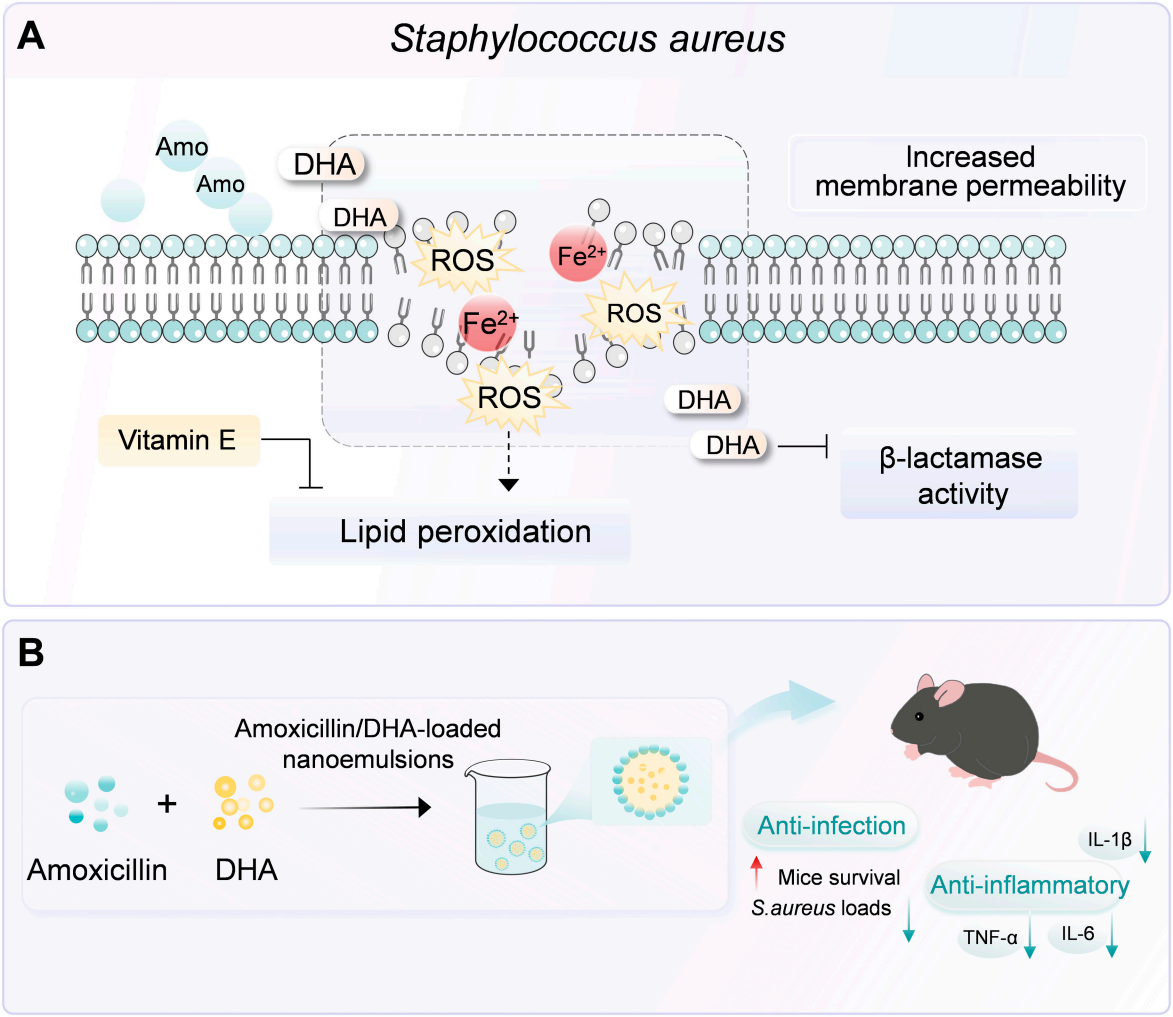
Proposed mechanism of synergistic interaction between DHA and β-lactam antibiotics. (A) DHA observably boosted the efficacy of β-lactam antibiotics against MRSA through the dual-faceted mechanisms of perturbation of lipid homeostasis and β-lactamase inhibition. (B) Importantly, to improve the bioactivity and stability, amoxicillin and DHA dual drug-loaded nanoemulsions (Amo/DHA-NEs) were prepared and characterized. As revealed, the combination therapy exerted the efficacious therapeutic potentials in vivo. This study elucidated the metabolic reprogramming mechanism behind the synergistic effect of DHA and β-lactam antibiotics and provided an effective approach to combat MRSA infections.

In summary, DHA was presented as a novel β-lactam adjuvant to potentiate β-lactam antibiotics activity by disrupting bacterial lipid homeostasis and inhibiting β-lactamase activity. This work revealed the potential of polyunsaturated omega-3 fatty acid DHA to combat drug-resistant bacterial infections and presented DHA and amoxicillin dual drug-loaded nanoemulsion as a promising and feasible therapeutic regimen (Fig. 8).

## Materials and Methods

### Bacterial strains

The MRSA strain USA300 was obtained from the American Type Culture Collection. *Escherichia coli* DH5a and *E*. *coli* BL21(DE3) cells were purchased from Sangon Biotech Co., Ltd. (Shanghai, China). The bacteria were respectively routinely grown and cultured in tryptone soy broth (TSB) or Luria-Bertani (LB) broth with shaking at 37 °C at 180 rpm. All bacterial culture media were purchased from Hope Bio-Technology Co., Ltd. (Qingdao, China).

### Reagents

All antibiotics were obtained from Macklin Biochemical Co., Ltd. (Shanghai, China) and Meilun Biotechnology Co., Ltd. (Dalian, China). DHA, AA, ALA, and EPA with a purity level of ≥98% were purchased from Derick Biotechnology Co., Ltd. (Chengdu, China).

### MIC determinations

The MICs of DHA and β-lactam antibiotics, including amoxicillin, ampicillin, the third-generation cephalosporins (ceftriaxone, cefixime, cefotaxime, and ceftazidime), the fourth-generation cephalosporins (cefpirome and cefepime), and other antibiotics (kanamycin, streptomycin sulfate, chloramphenicol, vancomycin, tetracycline, and erythromycin), against MRSA strain in the current study were determined using a standard broth microdilution method. In brief, the antimicrobial agents were diluted 2-fold in Mueller–Hinton broth (Hope Bio-Technology Co., Ltd., Qingdao, China) and mixed with bacterial suspensions (5 × 10^5^ CFUs/ml) in a germ-free 96-well microtiter plate. After 20-h incubation at 37 °C, the MICs were defined as the lowest concentrations of the compounds, with no visible bacterial growth. Results show that the synergistic activity was identified by FICI ≤ 0.50. FICI = (MIC_compoundA_ combination/MIC_compoundA_ alone) + (MIC_compoundB_ combination/MIC_compoundB_ alone). Additionally, the synergistic activities of PUFAs including EPA, ALA, and AA combined with amoxicillin were also conducted according to the above methods.

### Time-dependent killing and bacterial growth curves

In vitro time-killing curves were assessed to evaluate the synergistic efficacies of sub-MICs of β-lactam antibiotics (amoxicillin, cefixime, and cefpirome) alone, or in combination with DHA using MH broth with an initial inoculum of approximately 5 × 10^5^ CFU/ml MRSA cells. In addition, the inoculation of MRSA strain with the absence of agents served as the control. Further, at the indicated time point (0, 5, 10, and 24 h), each bacterial suspension was collected, diluted, and plated for microbial counting.

Additionally, the different concentrations of DHA and β-lactams (amoxicillin, cefixime, and cefpirome) alone or in combination were incubated with MRSA cultures at 37 °C. To determine the antibacterial activity of the combination of DHA plus β-lactam antibiotics, the absorbance values of the cultures at 600 nm were respectively measured at the indicated time points using a multifunctional Synergy H1 microplate reader (Biotek, USA).

### Cell viability determination

RAW264.7 cells or HeLa cells were cultured in 96-well plates with a cell density of 3 × 10^4^ cells per well. Then, increasing concentrations of DHA with the presence or absence of amoxicillin were added into the cell cultures for 6 h. After 6-h incubation, LDH released into the cell supernatant in different samples were detected using an LDH assay kit (Roche, Switzerland), according to the instruments.

### Metabolomics analysis

Metabolomics was conducted to provide insights into the potential biomarkers and enriched pathways underlying the synergistic activity of DHA in combination with amoxicillin. In brief, MRSA cells were treated with amoxicillin or the combination with DHA for a duration of 5 h. After centrifugation, the pellet cells were harvested and prepared for ultra-performance liquid chromatography–tandem mass spectrometry analysis to quantify the metabolite levels in MRSA under the diverse treatment. PCA, PLS-DA, and OPLS-DA were utilized to screen the key metabolites. Further, to identify the enriched biological pathways, the differential metabolite data were obtained by a multivariate statistical analysis and enriched pathway analysis was performed using Kyoto Encyclopedia of Genes and Genomes (KEGG) or the Human Metabolome Database (HMDB) database.

### Scanning electron microscopy

Cultivating MRSA in the logarithmic growth phase was collected and cultured with indicated concentrations of amoxicillin (64 μg/ml), DHA (16 μg/ml), and the combination. The bacterial suspensions with different treatment were subsequently subjected to incubation at 37 °C and 180 rpm for 5 h. After centrifugation (10,000 rpm, 10 min), the bacterial pellet was collected and washed with phosphate-buffered saline (PBS), and fixed with 2.5% glutaraldehyde. The bacterial structure and morphology under different treatments were observed and acquired using a scanning electron microscope (ZEISS SIGMA300, Germany).

### Live/dead fluorescence staining

Bacterial live/dead staining was employed to evaluate the antibacterial efficacy of amoxicillin, DHA monotreatment, and the combination treatment. Briefly, the logarithmic growth phase of MRSA was incubated with amoxicillin (64 μg/ml), DHA (16 μg/ml), and the combination for a duration of 5 h. The cells were collected and stained using the LIVE/DEAD BacLight Bacterial Viability Kit (Invitrogen, CA, USA). The images were captured by a confocal laser scanning microscope (OLYMPUS, IX73).

### Inner membrane permeability determination

PI was employed to assess the effect of different treatments on inner membrane permeability. Briefly, the MRSA cells (OD_600 nm_ = 0.5) were cultured with 0.5 μM PI (Sigma-Aldrich, USA) for 30 min, and the cells were incubated with different concentrations of DHA alone or in combination with amoxicillin (16, 32, and 64 μg/ml) for a duration of 1 h. Finally, the fluorescence intensity of the samples was measured with the excitation wavelength (Ex) at 535 nm and emission wavelength (Em) at 615 nm.

### Membrane depolarization assay

Membrane depolarization levels in diverse samples were monitored using DiSC_3_(5) dye (Sigma-Aldrich, USA). Indeed, cultivating MRSA in the logarithmic growth phase was collected and adjusted to an OD_600nm_ of 0.5. Further, the cultures were treated with DiSC_3_(5) (1.0 μM) for 10 min; subsequently, the cells were incubated with 8, 16, and 32 μg/ml DHA for an additional 50 min. The sample with the absence of DHA treatment served as the control. Finally, the fluorescence units were monitored with Ex = 622 nm/Em = 670 nm using a multifunctional Synergy H1 microplate reader (Biotek, USA). The effects of amoxicillin (32 and 64 μg/ml) alone or in combination of DHA (16 μg/ml) treatment on cell membrane depolarization were likewise measured according to the above procedures.

### Ferrous iron content analysis

Bacterial iron homeostasis was evaluated by measuring the intracellular ferrous iron content. The bacterial cultures were harvested after coincubation with indicated concentrations of DHA (0, 8, 16, and 32 μg/ml) or the combination of DHA plus amoxicillin. Then, the cells were lysed and the intracellular Fe^2+^ levels were determined by a Cell Ferrous Iron Colorimetric Assay Kit (Elabscience Biotechnology Co., Ltd., Wuhan, China), following the manufacturer’s instructions.

### CFU enumeration

The synergistic effect of DHA (16 μg/ml) in combination with amoxicillin (16 and 32 μg/ml) against *S. aureus* USA300 in the presence or absence of a ferrous iron chelator, 2,2’-bipyridine, or an antioxidant, vitamin E, was determined using time-dependent killing analysis. Briefly, the bacterial samples under different treatments were serially diluted and plated on LB agar plates at 0, 5, and 10 h.

### Oxidative stress and lipid peroxidation levels

The reactive oxygen species (ROS) levels in MRSA with the treatment of DHA alone or in combination with amoxicillin (16, 32, and 64 μg/ml) were determined using DCFH-DA (Beyotime Biotechnology, Shanghai, China) staining as previously described [35].

Further, hydroxyl radical formation was evaluated by a fluorescent dye, 3′-(p-hydroxyphenyl) fluorescein (HPF; Maokang Biotechnology Co., Ltd., Shanghai, China). Briefly, the MRSA cells (OD_600nm_ = 0.5) in the presence or absence of DHA, amoxicillin, or the combinations were harvested and then incubated with HPF (5 μM) for 30 min. The fluorescence units were quantized with the excitation wavelength at 492 nm and the emission wavelength at 515 nm.

The lipid peroxidation marker, MDA level in MRSA with the treatment of DHA alone or in combination with amoxicillin, was assessed using a Lipid Peroxidation MDA Assay Kit (Beyotime, Shanghai, China) according to the manufacturer’s instructions.

### Intracellular ATP level determination

Bacterial intracellular ATP levels in MRSA were determined using an Enhanced ATP Assay Kit (Beyotime, Shanghai, China). In brief, the bacterial suspension was centrifuged, washed, and incubated with diverse concentrations of DHA or in combination with amoxicillin for 1 h. Then, the bacterial cells were lysed and intracellular ATP contents were detected using a Synergy H1 microplate reader in the model of luminescence.

### qRT-PCR assay

A quantitative reverse transcription polymerase chain reaction (qRT-PCR) assay was performed to assess the oxidative stress-related gene transcriptional levels of MRSA with the presence of amoxicillin alone or in combination with DHA. Total RNA extracted from MRSA cells under different treatments was determined using a TRIzol-based method according to previous methods [36]. Reverse transcription to cDNA was performed using the StarScript III All-in-one RT Mix with gDNA Remover (GenStar, Beijing, China). Then, the messenger ribonucleic acid (mRNA) levels were determined by real-time PCR assays using 2× RealStar Universal SYBRqPCR Mix (GenStar, Beijing, China). The *16S* rRNA served as an internal control to normalize for transcript quantification, and the relevant primer sequences for qRT-PCR are listed in Table S1. The gene transcriptional levels in the different groups were calculated using the 2^−ΔΔ^Ct method.

### Expression and purification of the β-lactamase protein using the *E. coli* system

The *blaZ* gene, encoding β-lactamase, was amplified and synthesized entirely and incorporated into the pET28a expression vector. Further, the purification of the recombinant β-lactamase protein was conducted using affinity chromatography with the nickel-iminodiacetate resins according to previous methods [37].

### Inhibitory effect assessment of enzyme activity

The β-lactamase protein was initially incubated with different concentrations of DHA (0, 16, 32, 64, 128, and 256 μg/ml) for 1 h, followed by the addition of the substrate, nitrocefin (Aladdin, Shanghai, China), at a concentration of 0.2 mM. Then, the absorbance at 492 nm was measured to evaluate the inhibitory effect of DHA on β-lactamase catalytic activity.

### Molecular docking and molecular dynamics simulation

The receptor protein was downloaded from RCSB and then processed for molecular docking analyses with the small-molecule ligands, utilizing AutoDock Vina 1.1.2 software. Subsequently, the ligands were parameterized with the GAFF force field, and the protein was prepared using ff14SB force fields. The molecular dynamics simulation protocol comprised the following steps: energy minimization, heating, equilibration, and a production run. Finally, trajectory analysis involved computations of RMSD, RMSF, *R*_g_, and free energy calculations and profile analyses.

### Amo/DHA-NEs preparation and characterization

Amo/DHA-NEs were produced using WP as an emulsifier. In brief, 4% WP was resuspended in aseptic water and stirred. Amo/DHA-NEs were fabricated through high-energy homogenization, with 5% total oil phase (containing 20% DHA and an appropriate amount of medium-chain triglycerides) and aqueous emulsifier solution containing amoxicillin (20 mg/ml). Firstly, the system was emulsified at 15,000 rpm for 3 min utilizing a high-shear blender (IKA, Germany). Further, the coarse emulsions were homogenized using the Ultrahigh Pressure Nanometer Homogenizer (FB-110T2.6L, Shanghai, China) to produce nanoemulsions [14,38]. A TEM (HITACHI, H-7800, Japan) was employed to visualize the morphology of nanoemulsions after staining with phosphotungstic acid.

The encapsulation efficiency (EE%) and release profile were analyzed by high-performance liquid chromatography. In brief, EE calculation was carried out according to previous methods [12]. Further, the release experiments were conducted with the presence of trypsin. At the indicated time intervals, 1 ml of the sample was isolated and centrifuged, and the supernatant was collected for drug release determination. Meanwhile, 1 ml of buffer was used to mix the precipitate and added to maintain the volume of solution. To determine the DHA content released from nanoemulsions, DHA was extracted and the column employed for DHA analysis was a WondaSil C18 column (5 μm, 4.6  ×  250 mm) at a detection wavelength of 220 nm, with a flow rate of 1.0 ml/min. Solvent A [acetonitrile] and solvent B [H_2_O] were used as the mobile phases for gradient elution. In addition, the separation was performed using a C18 column (5 μm, 4.6  ×  250 mm) at a flow rate of 1.0 ml/min to detect the content of amoxicillin. The mobile phase was composed of 0.01 mol/l potassium dihydrogen phosphate buffer:methanol (90:10, v/v), and the detection wavelength was set at 225 nm. Ultimately, the standard curves were used to calculate the amoxicillin and DHA concentrations in nano-emulsions.

### Storage stability measurements

Amo/DHA-NEs nanoemulsions were respectively stored at temperatures of 4 and 25 °C for 7 days storage. The ZP, particle size, PDI, and morphology of the emulsions were recorded using Malvern Nano Zetasizer (Zetasizer Nano ZS90, UK).

### Simulated gastrointestinal digestion

Simulated gastric fluid (SGF) containing 10 mg/ml pepsin was mixed with the Amo/DHA-NEs at a mass ratio of 1:1, and then the mixture was adjusted to pH 2.5. To simulate the in vitro stomach digestion environment, the sample was shaken at 100 rpm for 2 h at 37 °C. At 0.5-h intervals (0, 0.5, 1, 1.5, and 2 h), the particle size and PDI were determined using Malvern Nano Zetasizer (Zetasizer Nano ZS90, UK).

Simulated intestinal fluid containing 10 mg/ml trypsin and 5 mg/ml bile salts was prewarmed and blended with the stomach digested solution in the above steps, at a mass radio of 1:1. Then, 0.4% sodium hydroxide (NaOH) was used to adjust to pH 6.8 and shaken at 100 rpm for an additional 2 h at 37 °C. The samples were harvested to measure the particle size and PDI at 0, 0.5, 1, 1.5, and 2 h postincubation.

### In vitro antibacterial properties of the nanoemulsions

Firstly, the MIC values of nanoemulsions against MRSA were determined using the standard broth microdilution method as previously described, and the optical density of samples at 600 nm was measured. Further, nanoemulsions were cocultured with MRSA at the early exponential phase (1 × 10^7^ CFU/ml) or late exponential phase (1 × 10^9^ CFU/ml). Then, the cultures were serially diluted and plated on LB agar plates at the indicated times (0, 5, and 10 h) and then photographed after 10-h incubation.

### Hemolysis rate evaluation

The erythrocytes at a final concentration of 5% (v/v) were mixed with the indicated concentrations of Amo/DHA-NEs at 37 °C for 1 h. The 0.1% Triton X-100-treated sample served as a positive control, and the PBS buffer-treated sample was used as a negative control. Then, the samples were centrifuged (10,000 rpm, 1 min), and the supernatants were collected and the absorbance of solutions was measured at 570 nm. The hemolysis ratio was calculated as follows: $\text{Hemolysis}\;\left(\%\right)=\left[\left(A_{570\;\text{samples}}-A_{570\;\text{negative control}}\right)/\right.\left.\left(A_{570\;\text{positive control}}-A_{570\;\text{negative control}}\right)\right]\times 100\%$.

### Animal studies

Female BALB/c mice or C57 BL/6 (6 to 8 weeks) mice were obtained from Liaoning Changsheng Technology Industrial Co., Ltd. All animal studies were approved and conducted in accordance with the guidelines of the Jilin University Institutional Animal Care Committee (SY202412076). All mice were housed in specific pathogen-free conditions (temperature = 23 ± 2 °C, humidity = 45% ± 10%).

### Safety evaluation of the nanoemulsions in vivo

SPF C57 BL/6 mice (6 to 8 weeks) were randomly divided into 3 groups: vehicle control group, low-dose treatment group, high-dose treatment group. Each group constituted a combined total of 6 mice. Subsequently, low-dose nanoemulsions (containing 20 mg/kg DHA and 40 mg/kg amoxicillin) and high-dose groups (containing 40 mg/kg DHA and 80 mg/kg amoxicillin) were respectively received at 12-h intervals for 3 consecutive days. The body weight and health conditions were recorded for 6 consecutive days. On day 6, blood samples were obtained for blood routine examination and liver, spleen, kidney, lung, heart, and intestine were collected for general observation, following euthanasia.

### Mouse systemic infection study

A mouse systemic infection model induced by the intraperitoneal injection of *S. aureus* was established. Briefly, C57 BL/6 mice were infected by intraperitoneal injection with MRSA USA300 at a dose of 2 × 10^8^ CFU, and the mice were randomly assigned to 5 groups (*n* = 6 per group): MRSA infection, DHA (20 mg/kg), amoxicillin (40 mg/kg), DHA in combination with amoxicillin (20 + 40 mg/kg), and Amo/DHA-NEs treatment (DHA, 20 mg/kg; amoxicillin, 40 mg/kg). The mice in the MRSA infection group were given an equivalent volume of the solvent, and the drugs were orally administered at 12-h intervals. Then, after 24 h postinfection, the mice were euthanized and the blood, kidney, spleen, and liver were collected for bacterial CFU analysis. Additionally, the kidney, spleen, liver, and intestine were fixed and paraffin-embedded for histopathological evaluation.

### MRSA-infected murine pneumonia model

A mouse model of acute MRSA pneumonia was further established to evaluate the therapeutical effect of amoxicillin in combination with DHA. In other words, MRSA cultures (5 × 10^8^ CFU) were dripped slowly into the trachea of anesthetized mice via the intranasal route of drug administration. The mice were randomly divided into 6 groups: control, MRSA infection, DHA (20 mg/kg), amoxicillin (40 mg/kg), DHA in combination with amoxicillin (20 + 40 mg/kg), and Amo/DHA-NEs treatment (20 mg/kg DHA and 40 mg/kg amoxicillin). The drugs were orally administrated at 12-h intervals. Subsequently, survival of mice in different groups was monitored and recorded daily.

Additionally, mice were infected by MRSA cultures (1 × 10^8^ CFU) and administered according to the above procedures. At 24 h postinfection, mice in different groups were euthanized. The lungs were harvested, ground, and diluted in the PBS medium and plated onto the TSB agar to analyze the MRSA burden in lungs. Moreover, histopathologic changes including hyperemia, edema, hemorrhage, degeneration, necrosis, and infiltration of inflammatory cells in the lungs were assessed by H&E staining. The contents of inflammatory cytokines (IL-1β, IL-6, and TNF-α) in lung tissue homogenates were analyzed using an ELISA (BioLegend, California, USA).

### MRSA-induced mastitis in mice

After 1 week of acclimatization feeding, female BALB/c mice were housed in individually ventilated cages and mated with male mice. Then, pregnancy was determined by the identification of vaginal plug, and the lactating mice after delivery were subsequently anesthetized. Next, the mice were infected with *S. aureus* USA300 (5 × 10^7^ CFU) by intraductal injection into the nipple of mice. The mice were randomly divided into 6 groups (*n* = 6 per group) and treated as described above. All the treatments were administered every 12 h. After 48 h postinfection, mice were respectively euthanized, and the mammary tissues were surgically harvested, ground into homogenates, and diluted for microbiological plating. Further, the mammary samples were fixed with paraformaldehyde and prepared paraffin sections, followed by the H&E staining. Pathological changes in the different groups were analyzed via microscopic examination and the histological changes were evaluated according to the previous criteria [39]. IL-1β and TNF-α levels in mammary tissue homogenates were detected and calculated by an ELISA, according to the manufacturer’s instructions.

### Statistical analysis

GraphPad Prism 8.0.2 was employed for data analysis and figure preparation. The data are expressed as means ± SD. Statistical significance level was calculated using 2-tailed unpaired Student’s *t* test or analysis of variance, and set as follows: *P < 0.05, **P < 0.01, and ns indicates no significance.

## Data Availability

The datasets produced in this study are available upon reasonable request.
