# Supplementary material for: Docosahexaenoic Acid-Driven Metabolic Reprogramming as an Attractive Anti-Infection Strategy to Potentiate β-Lactam Antibiotic Efficacy
Source: Research (Wash D C). 2025 Mar 28;8:0650. doi: 10.34133/research.0650 (PMC12692739; doi:10.34133/research.0650)
Supplement: Supplementary 1 — Table S1 Figs. S1 to S7 [file research.0650.f1.docx]

**DHA****-driven** **[metabolic reprogramming](https://pubmed.ncbi.nlm.nih.gov/39409945/)** **as an attractive anti-infection strategy to** **potentiate beta-lactam antibiotics efficacy**

Lei Xu^1^, Sangyu Hu^1^, Yuzhu Pei^1^, Yutong Zhou^1^, Xiuli Zhang^1^, Linlin Ding^1^, Minhe Cui^3^, Yonglin Zhou^2^, Xuming Deng^1^, Zihao Teng^1^*, Jianfeng Wang^1^*

^1^ State Key Laboratory for Diagnosis and Treatment of Severe Zoonotic Infectious Diseases, Key Laboratory for Zoonosis Research of the Ministry of Education, Institute of Zoonosis, and College of Veterinary Medicine, Jilin University, Changchun 130062, China.

^2^ Key Laboratory of Ministry of Education for Conservation and Utilization of Special Biological Resources in the Western China, School of Life Sciences, Ningxia University, Yinchuan, China.

^3^ Jilin Mushuo Breeding Co., Ltd, Changchun 130052, Jilin, China.

*Correspondence should be addressed to: Zihao Teng (tengzh7@outlook.com) or Jianfeng Wang ([wjf927@jlu.edu.cn](mailto:leixu22@jlu.edu.cn))

**Supplementary information**

| **Table S1. Sequence of primers used for** **qRT-PCR assays** | | |
| --- | --- | --- |
| **Gene** | **Primer** | **Sequence** |
| *BsaA_2* | Sense | GGGGACCCAAAGATTCCTGG |
|  | Anti-sense | AGGATCGTGGGTTTGTAGTGT |
| *katA* | Sense | \| CATGCCAAAGGTTCTGGTGC \| \| --- \| |
|  | Anti-sense | \| ATCCTCGAATGTCACGCTCC \| \| --- \| |
| *sodM* | Sense | \| CGCAGTGGGGCACTTTAGAT \| \| --- \| |
|  | Anti-sense | \| AGGCATGCTCCCAAACATCA \| \| --- \| |
| *sodA* | Sense | \| ATGCGCCAATGTAGTCAGGG \| \| --- \| |
|  | Anti-sense | CACGCTTTGGTTCAGGTTGG |
| *16s rRNA* | Sense | TGCCACCTACGTATTACCGC |
|  | Anti-sense | AGGTAACGGCTTACCAAGGC |

**
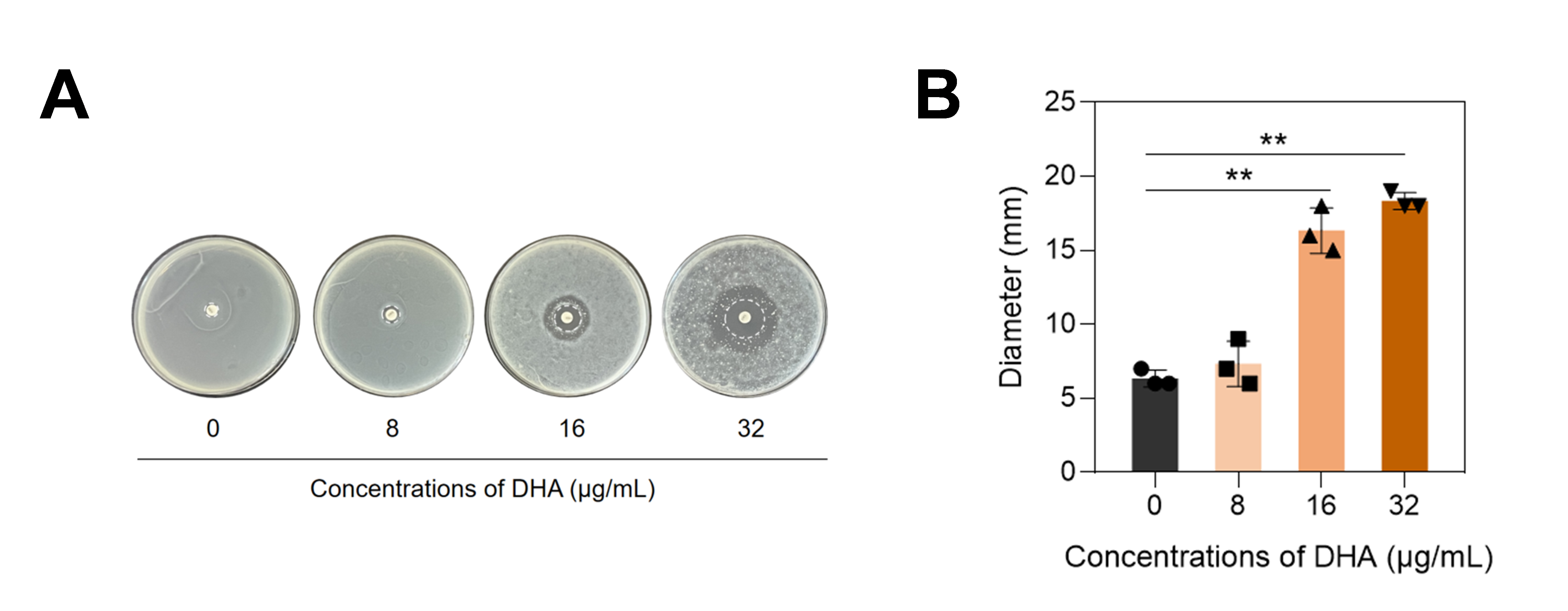
**

**Figure S1. Combined disk test of DHA in combination with amoxicillin against MRSA USA300**. The qualitative (**A**) and quantitative (**B**) results are representative of at least three biological replicates and all data were presented as mean ± SD. ***P* < 0.01.


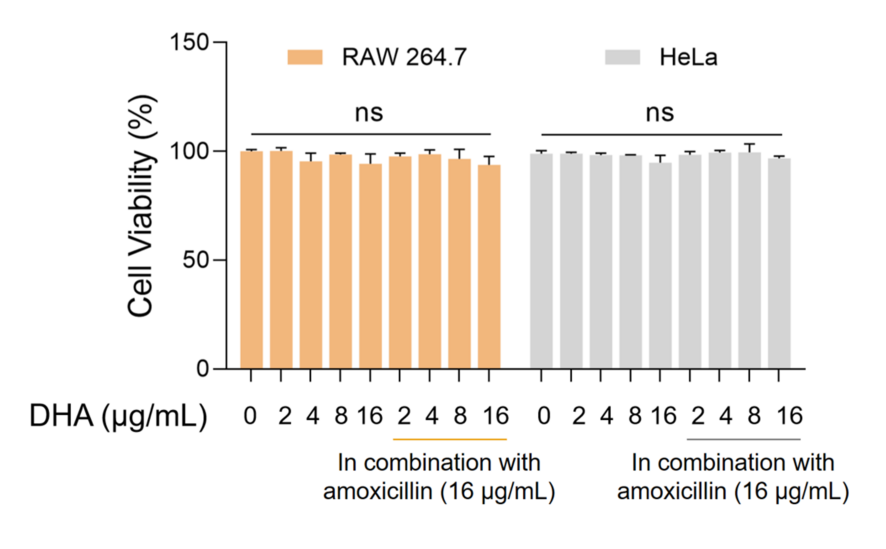


**Figure S2.** **Cell viabilities under the treatment of increasing concentrations of DHA with the presence or absence of amoxicillin were determined by LDH assay.** The experiments were repeated three times independently. ns, *P* > 0.05.

**
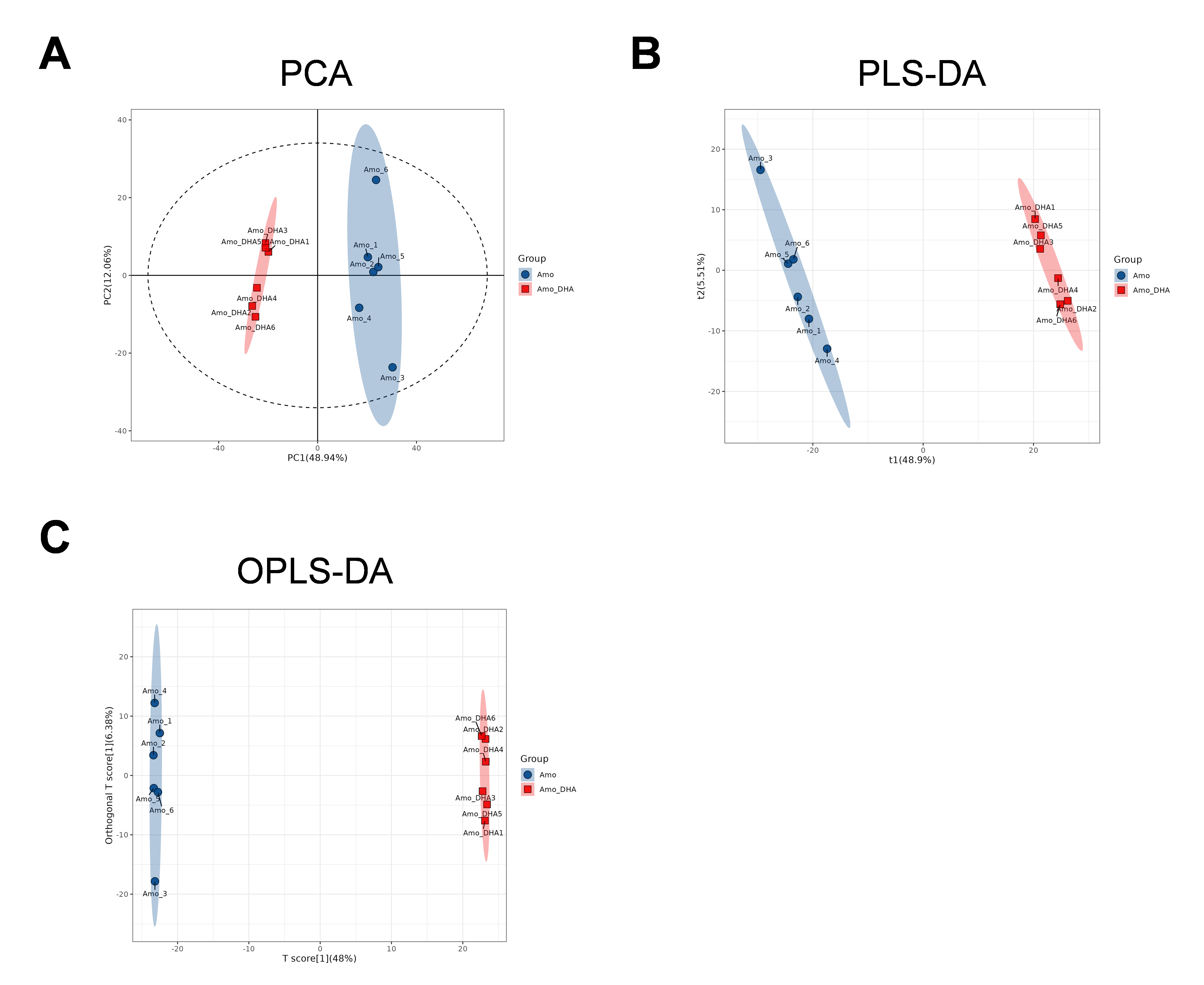
**

**Figure S3.** (**A-C**) Principal component analysis (PCA), partial least squares discrimination analysis (PLS-DA), orthogonal partial least squares discriminant analysis (OPLS-DA) models were established, showcasing the reliable data quality between amoxicillin monotreatment group and DHA+amoxicillin combination treatment group (n=6).


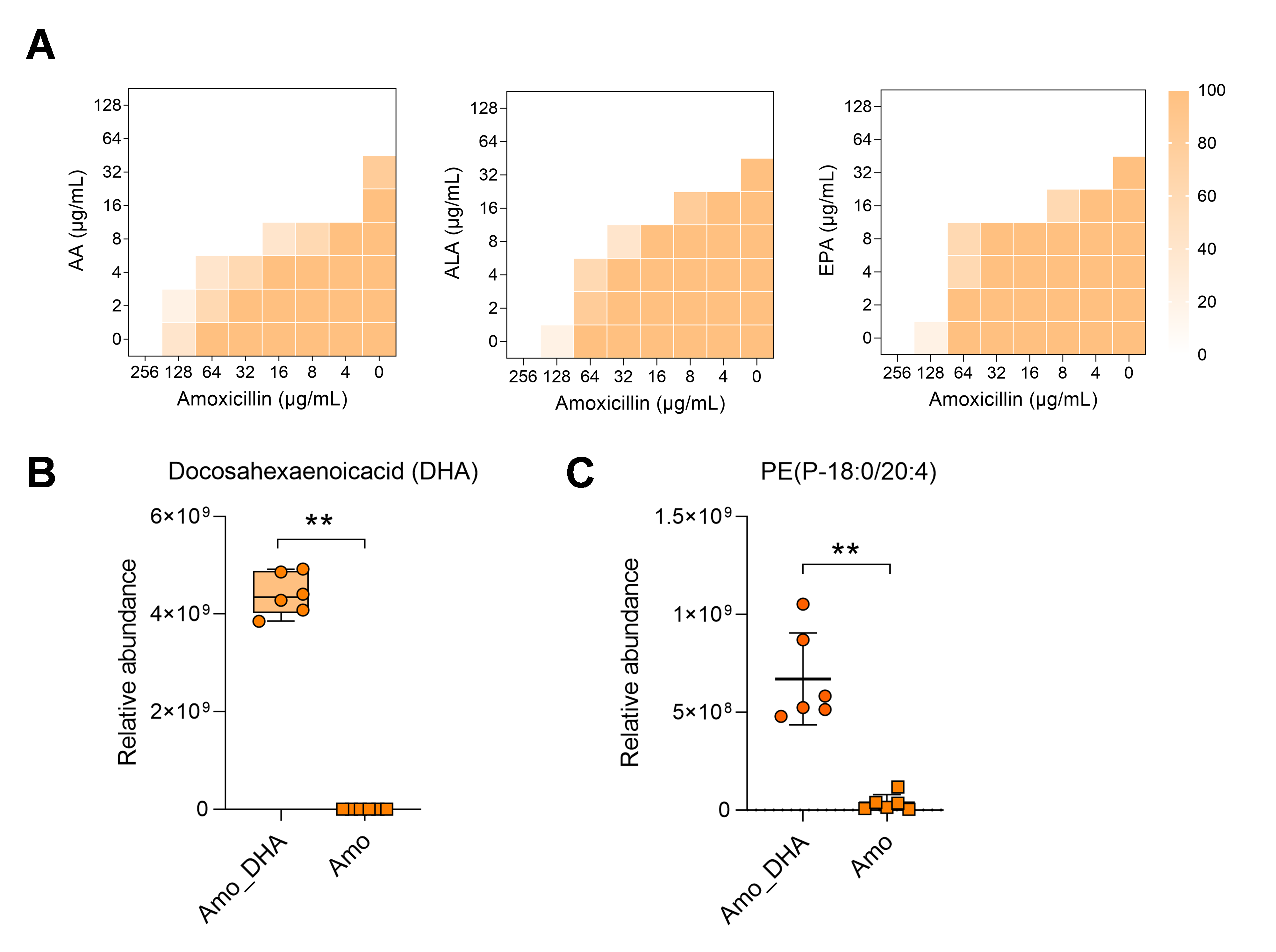


**Figure S4.** **PUFAs potentiate amoxicillin sensitivity in MRSA****.** (**A**) Chequerboard broth microdilution assays of arachidonic acid (AA), eicosapentaenoic acid (EPA) and alpha-linolenic acid (ALA) in combination with amoxicillin. Dark regions represent the higher cell density and were shown with orange color (n=5). (**B** and **C**) The relative expression levels of docosahexaenoic acid (DHA) and PE(P-18:0/20:4) in amoxicillin (Amo) and amoxicillin + DHA treatment (Amo_DHA) samples were determined (n =6). ***P* < 0.01.

**
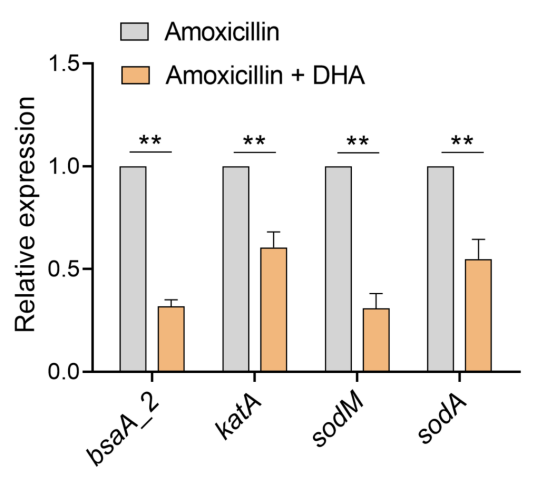
**

**Figure S5. qRT-PCR analysis of bacterial oxidative stress-related mRNA expressions.** Data are shown as means ± SD; n = 3 biological replicates. ***P* < 0.01.

**
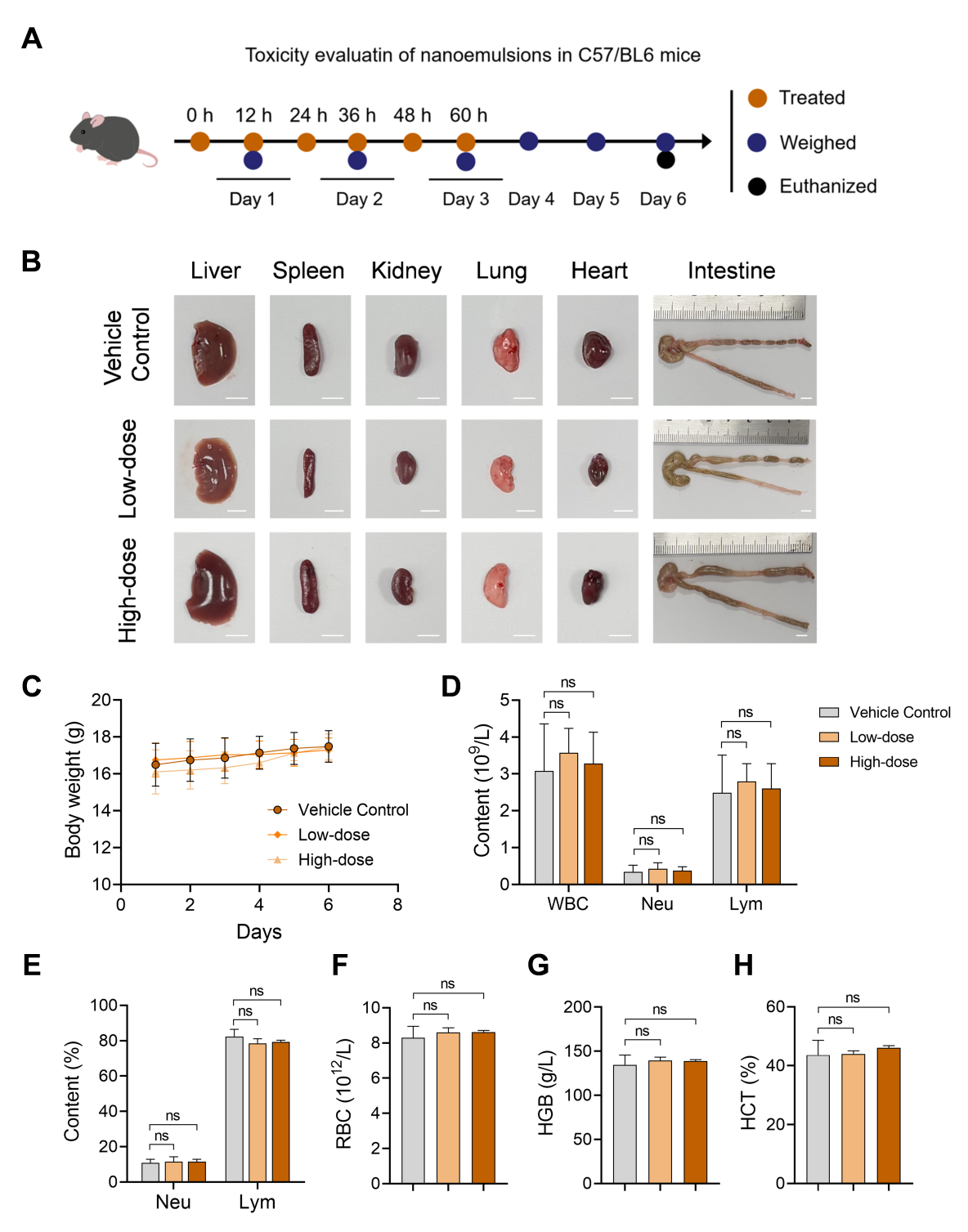
**

**Figure S6. Safety evaluation of** **Amo/DHA-NEs**. (**A**) The protocol of *in vivo* toxicity evaluation. (**B**) Gross autopsy of liver, spleen, kidney, lung, heart and intestine. Scale bar, 5 mm. (**C**) Body weight of mice under the different treatment (n=6). (**D-H**) Blood routine examination of mice in different groups (n=6). ns, *P* > 0.05.


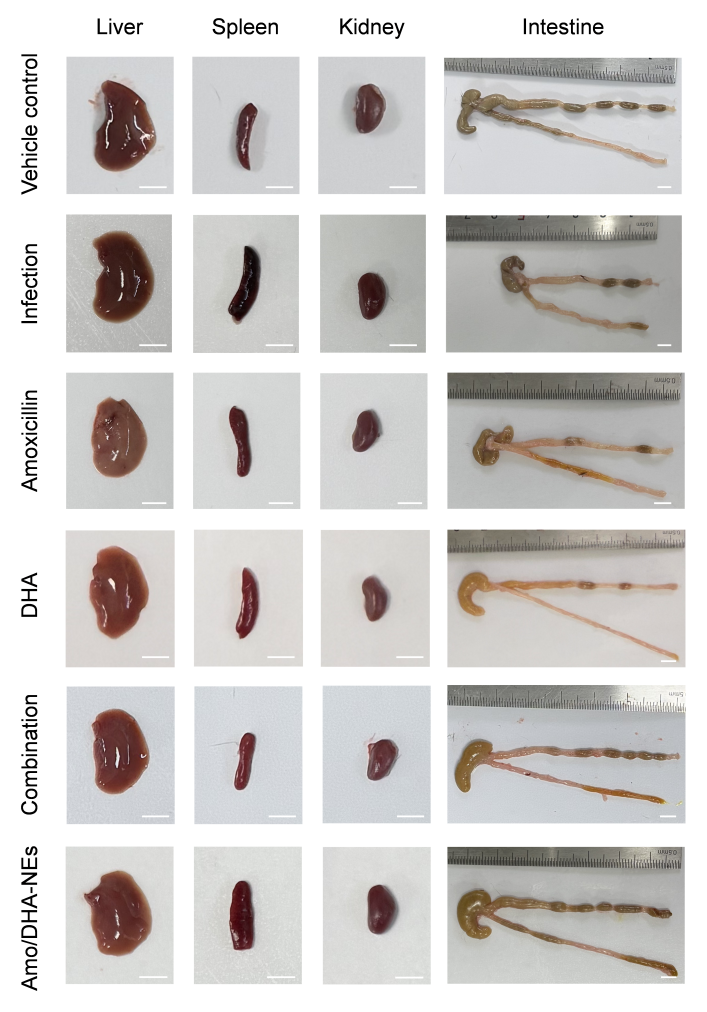


**Figure S7. *In vivo* efficacy evaluation in a mouse systemic infection model**. Gross autopsy of liver, spleen, kidney and intestine. Scale bar, 5 mm.
